# Valence bias arises from both positive and negative responses to ambiguous stimuli

**DOI:** 10.3389/fpsyg.2026.1832043

**Published:** 2026-08-26

**Authors:** Jordan E. Pierce, Nicholas R. Harp, James J. Gross, Maital Neta

**Affiliations:** 1 Department of Psychology, University of Nebraska-Lincoln, Lincoln, NE, United States; 2 Center for Brain, Biology, and Behavior, University of Nebraska-Lincoln, Lincoln, NE, United States; 3 Department of Psychology, University of California, Berkeley, Berkeley, CA, United States; 4 Department of Psychology, Stanford University, Stanford, CA, United States

**Keywords:** ambiguity, emotion, individual differences, unipolar, valence bias

## Abstract

**Background:**

Individuals vary in their tendency to interpret emotionally ambiguous stimuli as relatively more positive or negative, a trait-like difference known as “valence bias.” To date, research on valence bias has relied upon two-alternative forced-choice (2AFC) valence decisions that conflate the presence of positivity with the absence of negativity, and vice versa. This is problematic because more frequent negative judgments of ambiguous stimuli (i.e., a more negative valence bias) could represent true negative appraisals of those stimuli, or a difficulty in identifying a positive appraisal.

**Methods:**

In two preregistered studies, participants completed a standard 2AFC valence bias task (*n* = 100 and *n* = 508 in the respective studies) and provided separate positive and negative unipolar ratings of the same stimuli.

**Results:**

The 2AFC valence bias task captured independent effects of both positivity and negativity. Individuals with a more negative valence bias showed high endorsement of negativity *and* low endorsement of positivity; those with a more positive bias showed the opposite pattern. Individuals with a more positive valence bias also exhibited a stronger relationship between positive unipolar ratings and 2AFC categorization than individuals with a more negative valence bias, suggesting a greater sensitivity to positive appraisals. These results replicated in Study 2 in a larger, more diverse sample using unique stimulus sets.

**Discussion:**

Despite individual differences as a function of valence bias, ambiguous stimuli were endorsed as both positive and negative on unipolar scales, refuting claims that they might be “valence-free.” Collectively, these results demonstrated that multiple appraisals of ambiguity are recognized and weighted based on individual bias in valence processing during affective decision-making.

## 1 Introduction

Valence—whether a stimulus is perceived as positive or negative—is a core factor driving human behavior, including approach-avoidance motivation, learning, and decision-making (Barrett, 2006; Cacioppo et al., 2011; Jäger et al., 2020; Schechtman et al., 2010; Shuman et al., 2013). Some stimuli convey a relatively clear positive or negative valence and yield consistent interpretations across individuals—most people agree that spending time with a loved one is positive/rewarding, but sustaining a physical injury is negative/harmful. Other stimuli, however, have a more ambiguous valence, meaning that they can be interpreted as either positive or negative by different individuals and they evoke a wider range of emotional responses between individuals—some people might interpret an image of two people in a tearful embrace as sorrowful (negative), while other people interpret the same event as a joyful reunion (positive). Critically, individuals differ in their tendency to interpret emotionally ambiguous stimuli as relatively more positive or negative, a difference known as “valence bias” (Harp et al., 2021; Neta et al., 2009). Evidence suggests that, in young adults, the initial evaluation of ambiguity tends to be negative (Neta and Whalen, 2010). In contrast, a more positive bias is associated with longer response times (Neta and Tong, 2016), possibly associated with reappraising stimuli to frame them in a positive light (Mauss et al., 2007; Neta et al., 2023). Although an initial negative judgment of ambiguous stimuli may provide an advantage in allowing quick reactions to avoid potential threats, the persistence of a negative valence bias could lead to maladaptive psychological outcomes (Brock et al., 2022; Brown et al., 2017; Park et al., 2016; Petro et al., 2021b).

### 1.1 The impact of measurement choice on affective responses

Prior research on valence bias has mostly relied upon two-alternative forced-choice (2AFC) valence decisions (positive vs. negative). The main advantage of this approach is that the binary categorization forces individuals to reveal their internal bias toward ambiguity, rather than allowing them to classify ambiguity as its own category or avoid an effortful decision by classifying ambiguity as neutral (i.e., neither positive nor negative). Binary responses can also be made quickly, emphasizing an individual’s gut reaction while limiting the influence of elaborated cognitive processing. The disadvantage of this approach, however, is that it confounds greater positivity with lower negativity (and vice versa) by allowing only a single response that reflects the sum of any valence analysis, without probing how this decision is reached. Yet there are many ways to assess positive and negative valence, including 2AFC decisions, bipolar dimensions (Russell and Carroll, 1999; Watson and Tellegen, 1985), unipolar scales (Kron et al., 2015; Schimmack, 2005; Watson and Clark, 1997), or a multidimensional combination of scales (e.g., the evaluative space grid, Larsen et al., 2009), that may influence how individuals report valence appraisals.

When faced with ambiguity, individuals may focus on only the positive features, only the negative features, or both to make their 2AFC decision. For example, an individual might simultaneously recognize both the positive and negative interpretation of the tearful embrace mentioned above. Yet if they have a general negativity bias (i.e., weighing negative features more strongly than positive features; Baumeister et al., 2001; Norris, 2021; Rozin and Royzman, 2001), they may ultimately judge the scene as negative in a 2AFC task. Alternatively, another individual may focus only on negative interpretations of the scene, giving no thought to positivity, but their 2AFC response would be identical to the individual who considers both sides. In other words, unipolar evaluations of positivity and negativity should contribute to an individual’s 2AFC judgment, but positivity and negativity may be weighted differently.

In the standard 2AFC task used in prior work, it was impossible to determine the extent to which positive and negative appraisals of valence contributed to the response outcome or how intense these appraisals were. Existing literature, therefore, cannot provide evidence as to whether the link between a negative valence bias and mood disorders, for example, is driven more by an excess of negativity or by the absence of positivity. Furthermore, some researchers have argued that mixed 2AFC responses to ambiguous stimuli, such as surprised expressions, mean that they are unvalenced (*neither* positive nor negative; Ortony, 2022) and, therefore, that surprise is not an emotion. Assessing ambiguous stimuli with unipolar positive and negative scales should demonstrate the extent to which individuals endorse *both* positive and negative valence in these stimuli (or fail to do so).

### 1.2 The current study

In the current work, we sought to fill this gap in the literature on emotional ambiguity and disentangle the relative contributions of positivity and negativity to valence bias by comparing the standard valence bias task that utilizes 2AFC decisions to a version with unipolar response scales. In two preregistered online studies, participants completed the standard 2AFC valence bias task and also rated the stimuli on positive and negative unipolar scales (in separate blocks). We hypothesized that 2AFC valence bias would be related to both positive and negative unipolar ratings of ambiguous emotional stimuli, such that a positive valence bias would be associated with both high positivity and low negativity and a negative bias would be associated with both high negativity and low positivity. We also predicted that, overall, participants would appraise ambiguous stimuli as both positive and negative to some extent (versus *neither* positive nor negative). The first study tested these hypotheses in a relatively small sample using stimuli consisting of positive, negative, and ambiguous faces, scenes, and words; the second study extended these findings to a larger, more diverse sample and included a new set of faces and scenes to improve generalizability of the findings. Together, these studies demonstrated how positive and negative unipolar appraisals contribute to valence judgments of ambiguity and inform our understanding of how affective interpretations are generated.

## 2 Study 1: 2AFC vs. unipolar ratings

### 2.1 Materials and methods

#### 2.1.1 Participants

Participants were recruited via Amazon’s MTurk online platform in 2022. Participants were eligible if they were at least 18 years old, a native English speaker, and had no history of psychological or neurological disorders. The task was only accessible via computer (i.e., no phones or tablets) to participants in the United States. All participants provided informed consent prior to completing a demographic screener that assessed the three inclusion criteria. All participants who completed the eligibility screener (*n* = 216), even those who were ultimately excluded, received monetary compensation; eligible participants that completed the full study (*n* = 122) received additional compensation. All procedures were approved by the local institutional review board.

As defined in the preregistration, recruitment continued until 100 participants with usable data in the 2AFC task were collected (additional participants were excluded for the unipolar task, see Results). An *a priori* power analysis (G*Power) indicated a necessary sample size of at least 97 participants for a bivariate correlation ( α = 0.05; power = 80%) to detect a small effect size (r = 0.25; Faul et al., 2007). Of the 216 participants who began the study, five were excluded based on their responses to the initial demographic screener and 89 were excluded for failing to complete the full study within the time limit (90 min). Of the 122 participants who completed the full study, 17 were excluded for inaccurate responses (<60% accuracy for positive/negative stimuli) in the 2AFC task, as in prior work (Harp et al., 2021; Neta and Tong, 2016), and 5 were excluded for failed attention checks. The remaining 100 participants consisted of 31 men, 67 women, and 2 non-binary individuals, aged 21–69 years (mean (SD) = 38.9 years (10.9)), who reported their race as 6 Asian/Asian American, 12 Black/African American, 77 White/European American, 5 Other/Mixed, and their ethnicity as 14 Hispanic/Latinx and 86 not Hispanic/Latinx.

#### 2.1.2 Stimuli

Stimuli included emotional facial expressions, scenes, and words, consisting of clearly valenced (positive, negative) and ambiguous trials (Brock et al., 2022; Neta et al., 2009). Faces expressed happiness, anger, or surprise, while scenes and words encompassed a broader range of emotions such as fear, disgust, or love with ambiguous examples that could be interpreted variously. More specifically, ambiguous stimuli were selected for having a roughly equal probability of being evaluated as positive or negative and previously exhibited high disagreement in valence ratings/categorizations across people (Harp et al., 2021; Neta et al., 2013). This included, for example, an image of a group of clowns or the word RETREAT (see Supplementary Table S1 for a complete list of stimuli). Positive and negative valence in this task, therefore, was operationalized as a general pleasantness or unpleasantness that was expressed or elicited by the stimulus as evaluated by the participant.

Twelve sample images (four each of faces, scenes, and words) were presented prior to each iteration of the task as a practice block to ensure that participants understood the task instructions. Next, six task blocks were presented in each iteration of the task. Of these six blocks, there were two blocks each of faces, scenes, and words with 24 ambiguous and 24 clear images. Altogether, there were 48 faces, scenes, and words presented in each iteration of the task, totaling 144 trials.

The face stimuli were selected from the NimStim (Tottenham et al., 2009) and Karolinska Directed Emotional Faces (Lundqvist et al., 1998) databases, the scenes were selected (Neta et al., 2013) from the International Affective Picture System (Lang et al., 2008), and the words were taken from a set identified in prior work (Harp et al., 2021). Faces and scenes were presented in color and words were presented in capital letters in black font in the center of a white background (for more details, see Harp et al., 2021).

#### 2.1.3 Procedure

All tasks were administered using Gorilla Experiment Builder (Anwyl-Irvine et al., 2020). Participants first completed the eligibility screener described above, followed by three different iterations of the task that varied in response format: (1) two-alternative forced choice categorizations (2AFC; “positive” versus “negative”), (2) unipolar ratings of positive valence (“1—Not at all positive” through “5—Extremely positive”), and (3) unipolar ratings of negative valence (“1—Not at all negative” through “5—Extremely negative”). All participants in Study 1 completed the 2AFC version first, and the order for the unipolar tasks was counterbalanced across participants. Prior to each task version, participants were instructed to respond as quickly and accurately as possible and completed practice trials to familiarize them with the response buttons. For the unipolar version, participants were informed beforehand that there would be separate positive and negative ratings made during the task.

For each iteration of the task, participants were randomly assigned to a pseudorandom order of the face, scene, and word blocks. For the 2AFC task, participants used the “A” and “L” keys on the keyboard to respond (counterbalanced across participants). For the unipolar ratings, participants used the 1, 2, 3, 4, and 5 keys to respond. All stimuli were preceded by a 1,500 ms fixation cross and presented for 500 ms; participants had 2,000 ms to respond.

#### 2.1.4 Analyses

Valence bias was calculated as the mean percent of negative categorizations across all stimulus classes (faces, scenes, words) of the ambiguous stimuli in the 2AFC task. Similarly, average unipolar positive ratings and average negative ratings were calculated from the mean ratings across all stimulus classes. Trials with reaction times (RTs) faster than 250 ms were excluded as too fast to reflect typical perception and decision-making times (Harp et al., 2021; Whelan, 2008). Repeated-measures ANOVAs were conducted on average valence bias and unipolar ratings to confirm valence differences between positive, negative, and ambiguous stimuli, with Greenhouse–Geisser corrected degrees of freedom for violation of the sphericity assumption. The Spearman’s rank correlation between unipolar positive and negative ratings was examined across participants. Additionally, the minimum value between a participant’s response for the unipolar positive and negative rating for each stimulus (Larsen et al., 2017) was averaged for each valence category (positive, negative, ambiguous). This measure was explored to determine whether ambiguous stimuli showed higher minimum unipolar ratings (either positive or negative) than positive stimuli (which should have low negative ratings) or negative stimuli (which should have low positive ratings). Finally, a linear model predicting 2AFC valence bias was fit with positive and negative unipolar ratings as predictors; age, sex, race/ethnicity (Asian, Black, Hispanic, White, Mixed/Other), and unipolar task order (positive/negative ratings first) were included as covariates.

As described in the preregistration, participants completed several self-report questionnaires on psychological ill-being, well-being, and emotion regulation, which were analyzed for correlations with both 2AFC valence bias and unipolar ratings. Surveys included the Beck Depression Inventory (Beck et al., 1961), Cognitive Flexibility Scale (Martin and Rubin, 1995), Emotion Beliefs Questionnaire (Becerra et al., 2020), Emotion Regulation Questionnaire (Gross and John, 2003), Eudaimonic Well-being scale (Ryff and Keyes, 1995), Peace of Mind scale (Lee et al., 2013), Primals Inventory (Clifton and Yaden, 2021), PROMIS Anxiety scale (Pilkonis et al., 2011), Satisfaction with Life Scale (Diener et al., 1985), and the Scale of Positive and Negative Experience (Diener et al., 2010). Details on the questionnaires and related non-significant results are provided in the Supplementary material.

We report how we determined our sample size, all data exclusions, manipulations, and measures. Data are available on the Open Science Framework.^1^ All analyses were conducted and figures created in R Statistical Software, version 4.2.3 (R Core Team, 2023). This study was preregistered at AsPredicted.org (#100002; https://aspredicted.org/blind.php?x=MTB_PTR).

### 2.2 Results and discussion

#### 2.2.1 2AFC task

Response categorizations (positive or negative) for each stimulus class (faces, scenes, and words) in the 2AFC task were summarized as the percentage of negative responses. Details are provided in Table 1. As expected, average responses for positive stimuli were lower (more positive) than ambiguous stimuli, which were lower than negative stimuli (*F*(1.83, 180.83) = 1970.84, *p* < 0.001, η = 0.952), demonstrating that the assigned stimulus valence labels were appropriate.

**Table 1:** Average responses in the 2AFC (percent negative) and unipolar tasks (1–5 rating).

Task	Stimuli	Positive stimuli		Ambiguous stimuli		Negative stimuli	
		Median (IQR)	Range	Median (IQR)	Range	Median (IQR)	Range
Study 1 (*n* = 100 2AFC, *n* = 75 Unipolar)							
2AFC	Faces	8.33 (16.67)	0–36.36	79.17 (35.14)	0–100	91.67 (8.33)	60.00–100
	Scenes	0.00 (8.33)	0–33.33	41.67 (15.94)	8.33–75.00	90.00 (16.67)	63.64–100
	Words	0.00 (8.33)	0–25.00	58.33 (26.09)	4.17–95.83	100.00 (8.33)	75.00–100
	Total	5.05 (8.33)	0–33.18	58.91 (20.70)	5.93–91.10	91.67 (9.29)	73.48–100
Unipolar positive	Faces	4.58 (0.91)	2.80–5.00	2.57 (1.04)	1.00–4.30	1.50 (0.83)	1.00–3.33
	Scenes	4.75 (0.63)	2.75–5.00	2.92 (0.62)	1.88–4.55	1.50 (0.58)	1.00–3.17
	Words	4.73 (0.58)	3.50–5.00	2.63 (0.81)	1.58–4.92	1.33 (0.42)	1.00–3.17
	Total	4.64 (0.51)	3.38–5.00	2.74 (0.62)	1.52–4.49	1.44 (0.52)	1.00–2.97
Unipolar negative	Faces	1.33 (0.83)	1.00–3.64	2.92 (0.91)	1.08–4.83	4.17 (1.14)	1.92–5.00
	Scenes	1.33 (0.58)	1.00–3.33	2.75 (0.67)	1.63–4.33	4.17 (0.78)	2.25–5.00
	Words	1.18 (0.45)	1.00–3.75	3.09 (0.72)	1.13–4.30	4.67 (0.79)	2.17–5.00
	Total	1.33 (0.47)	1.00–3.25	2.88 (0.45)	1.58–3.86	4.28 (0.79)	2.47–4.94
Study 2 (*n* = 508 2AFC, *n* = 460 Unipolar)							
2AFC	Faces	8.33 (16.67)	0–40.00	70.83 (41.67)	0–100	91.67 (16.67)	60.00–100
	Scenes	8.33 (9.09)	0–40.00	50.00 (25.00)	4.17–91.67	91.67 (16.67)	60.00–100
	Words	0.00 (8.33)	0–36.36	60.87 (28.08)	4.17–100	100.00 (909)	60.00–100
	Total	5.56 (9.72)	0–35.42	59.18 (25.76)	7.34–97.83	91.67 (11.62)	63.07–100
Unipolar positive	Faces	4.42 (0.92)	2.00–5.00	2.79 (0.76)	1.00–5.00	1.92 (1)	1.00–4.00
	Scenes	4.33 (0.83)	2.27–5.00	2.96 (0.79)	1.09–4.63	1.67 (0.85)	1.00–3.78
	Words	4.67 (0.67)	2.33–5.00	2.67 (0.75)	1.13–4.45	1.41 (0.67)	1.00–3.67
	Total	4.44 (0.72)	2.25–5.00	2.79 (0.58)	1.42–4.30	1.69 (0.67)	1.00–3.44
Unipolar negative	Faces	1.50 (0.83)	1.00–4.25	2.91 (0.92)	1.00–5.00	3.83 (1.21)	1.55–5.00
	Scenes	1.42 (0.74)	1.00–4.25	2.69 (0.72)	1.00–4.74	3.83 (1.17)	1.67–5.00
	Words	1.33 (0.73)	1.00–4.10	3.00 (0.75)	1.04–4.79	4.33 (0.85)	1.70–5.00
	Total	1.42 (0.69)	1.00–4.17	2.83 (0.64)	1.10–4.22	3.97 (0.89)	1.70–5.00

#### 2.2.2 Unipolar task

For the unipolar task, several thresholds (1, 2, or 3 SD) were considered for excluding participants based on their responses to positive and negative stimuli, as described in the preregistration. While there are no “correct” ratings for the unipolar task (i.e., even a clearly negative image can be rated as mildly or extremely negative), average ratings that are outliers relative to the group distribution may suggest that a participant failed to understand the task or the response options. If outliers were defined as more than 1 SD from the mean of either the positive or negative scale, 59 out of the 100 participants were excluded. If outliers were defined as more than 2 SD from the mean, 25 participants were excluded. If outliers were defined as more than 3 SD from the mean, only one participant was excluded. Accordingly, a 2 SD threshold was selected as the most reasonable criterion to ensure data quality while retaining a sufficient sample size. (Given the data-driven nature of this thresholding approach, models relating 2AFC and unipolar task responses at the alternative SD cutoffs were also tested, remained significant, and are provided in the Supplementary material). The final sample consisted of 75 individuals: 26 men, 47 women, and 2 non-binary individuals, aged 22–69 years [mean (SD) = 38.17 (10.42)] who reported their race as 5 Asian, 10 Black, 55 White, 5 Other, and their ethnicity as 12 Hispanic/Latinx and 63 not Hispanic/Latinx.

Average ratings across participants for each stimulus class and valence are provided in Table 1 (see also Supplemetnary Figure S1). Overall ratings for the positive and negative scales were calculated as the average of the responses for the three stimulus classes (faces, scenes, and words). As with the 2AFC task, average unipolar positive ratings were higher for positive stimuli than ambiguous stimuli, which were higher than negative stimuli (*F*(1.59, 117.85) = 761.22, *p* < 0.001, η = 0.911). Conversely, average unipolar negative ratings for positive stimuli were lower than ambiguous stimuli, which were lower than negative stimuli (*F*(1.59, 117.51) = 666.82, *p* < 0.001, η = 0.900). Participants’ positive and negative unipolar ratings of ambiguous stimuli were negatively correlated across all stimulus classes ( ρ  = − 0.28, 95% CI [−0.51, −0.01], *p* = 0.016) and for each class separately (faces: ρ  = − 0.30, 95% CI [−0.53, −0.04], *p* = 0.008; scenes: ρ  = − 0.34, 95% CI [−0.55, −0.07], *p* = 0.003; words: ρ  = − 0.41, 95% CI [−0.61, −0.16], *p* < 0.001), indicating that the scales were partially treated as opposites, yet also captured unique variance for positivity and negativity.

Next, the minimum value of the unipolar positive or negative rating for each stimulus within a participant was averaged for each valence category. For positive stimuli, the average minimum rating was 1.52 (SD = 0.47) and for negative stimuli, the average minimum rating was 1.53 (SD = 0.41). For ambiguous stimuli, the average minimum rating was 1.97 (SD = 0.40), which was significantly higher than either positive (*t*(222) = 6.33, *p* < 0.001) or negative (*t*(222) = 6.38, *p* < 0.001) valence stimuli.

#### 2.2.3 2AFC vs. unipolar responses

To compare responses in the 2AFC and unipolar tasks, a regression model was fit predicting 2AFC valence bias from unipolar positive and negative ratings, using participants’ average ratings across all stimulus classes (i.e., ambiguous faces, scenes, and words). Age, sex, race/ethnicity, and unipolar task order (negative or positive ratings first) were included as covariates. The model was significant overall (*F*(9, 65) = 7.657, *p* < 0.001, *R*^2^ = 0.51) with both unipolar positive and unipolar negative ratings explaining significant variance in 2AFC valence bias (Table 2, Supplementary Figure S2). A post-hoc power analysis indicated that with *n* = 75, R^2^ = 0.51, and 9 predictors, the linear regression had power >99% to detect a significant model. In addition, a model with only the covariates as predictors was not significant (*F*(7, 67) = 1.036, *p* > 0.05, *R*^2^ = 0.10), such that the change in variance explained by the full model with unipolar ratings was $\Delta R^{2}$ = 0.42. Models considering each stimulus class separately also showed significant effects of unipolar ratings (see Supplementary Tables S3–S5).

**Table 2:** Model predicting 2AFC valence bias from unipolar positive and negative ratings in Study 1.

Predictor	*b* (SE)	*t*	*p*	95% CI	*r_a(b.c)_*
Intercept	**68.81 (12.83)**	**5.37**	< 0.001**	**[43.20, 94.42]**	
Unipolar negative	**9.19 (2.88)**	**3.19**	0.002**	**[3.43, 14.95]**	**0.275**
Unipolar positive	**−15.10 (2.65)**	**−5.70**	< 0.001**	**[−20.40, −9.81]**	**−0.521**
Age	0.21 (0.14)	1.52	0.135	[−0.07, 0.48]	0.120
Sex (male)^a^	−1.05 (2.96)	−0.36	0.724	[−6.96, 4.86]	−0.045
Race (black)^b^	−5.16 (7.22)	−0.72	0.477	[−19.59, 9.26]	−0.071
Race (hispanic)^b^	−1.50 (6.64)	−0.23	0.822	[−14.76, 11.76]	
Race (mixed/other)^b^	5.10 (8.72)	0.59	0.561	[−12.32, 22.52]	
Race (white)^b^	−4.99 (6.05)	−0.83	0.413	[−17.08, 7.10]	
Unipolar task order^c^	−0.15 (2.68)	−0.06	0.954	[−5.50, 5.19]	0.011

a Reference = Female.

b Reference = Asian.

c Reference = negative ratings first.

r_a(b.c)_ = semi-partial correlation. Bold font indicates a significant effect: **p < 0.01.

#### 2.2.4 Summary

In this study, participants completed 2AFC valence categorizations based on their appraisals of clearly positive, clearly negative, and ambiguously valenced stimuli. They subsequently provided positive and negative unipolar ratings for the same stimuli. Results revealed that the 2AFC valence bias task captured independent effects of both positivity and negativity. Individuals with a more negative valence bias showed low unipolar ratings of positivity and high unipolar ratings of negativity, while those with a more positive 2AFC valence bias showed the opposite pattern. These results demonstrate that individual differences in valence judgments are associated with both the positive and negative features that may be perceived in a stimulus, with participants recognizing multiple possible interpretations as valid.

Additionally, an exploration of the average minimum unipolar rating for each stimulus within a participant demonstrated that ambiguous stimuli had a higher minimum than either positive or negative stimuli. This means that while individuals provided low minimum ratings for clearly valenced stimuli (e.g., positive stimuli had low unipolar negative ratings), indicating strong differentiation of positive and negative valence, ambiguous stimuli yielded at least partial endorsement of both positivity and negativity for the same stimulus. These mixed ratings for ambiguous stimuli demonstrate that dual-valence interpretations of ambiguity are occurring within an individual, not merely across subjects with different appraisals of the stimulus.

## 3 Study 2: replication in a larger sample with diverse stimuli

The findings from Study 1 demonstrated initial support for our predictions about the association between 2AFC valence bias and unipolar ratings of positivity and negativity for ambiguous stimuli. However, the sample was of modest size and consisted of predominantly White participants, the stimuli included only White faces, and the scenes were selected from a single, older image database. In a preregistered follow-up study, we sought to collect a larger, more racially diverse sample to assess the generalizability of Study 1’s findings. In addition, we substituted the face stimuli with a newer, racially diverse set and the scene stimuli with newer images that were assigned positive, negative, and ambiguous labels based on pilot data using the 2AFC task. Finally, given the possibility of task order influencing responses (Adams et al., 2016; Chaxel et al., 2016), we counterbalanced whether participants completed the 2AFC or unipolar task first in Study 2. Initial analyses were identical to Study 1 and additional exploratory analyses were included to follow-up on potential effects of interest in this larger sample. We expected the results from Study 2 to replicate Study 1 and to demonstrate that the relationship between unipolar ratings and a forced choice categorization extends across a broader set of contexts.

### 3.1 Materials and methods

#### 3.1.1 Participants

Participants were recruited via the Prolific online platform^2^ in 2024. Participants were eligible if they were between 18 and 65 years old and had no history of psychological or neurological disorders. The task was only accessible via computer (i.e., no phones or tablets) to participants in the United States. All participants provided informed consent, then completed a demographic screener for the above exclusion criteria and to ensure balanced race/ethnicity groups (see below). Eligible participants that completed the full study (*n* = 623) received monetary compensation. All procedures were approved by the local institutional review board.

As defined in the preregistration, recruitment continued until approximately 100 participants in each of four target race/ethnicity groups were collected who had usable data for both tasks. These groups consisted of participants who identified as Hispanic/Latinx (regardless of race), Asian/Asian American (non-Hispanic), Black/African American (non-Hispanic), and White/Caucasian/European American (non-Hispanic). An *a priori* power analysis (G*Power) indicated a necessary sample size of 100 participants for an independent *t*-test between groups ( α = 0.05; power = 80%) to detect an effect size of d = 0.4 (Faul et al., 2007). Race/ethnicity intergroup effects will be not considered in the current analysis but rather addressed in full elsewhere.

Of the 623 participants who completed the full study, 115 were excluded for inaccurate responses (<60% accuracy for positive/negative stimuli) or low trial counts (<75% trials) in the 2AFC task (remaining *n* = 508), and 167 were excluded for inaccurate responses [>2 SD from the sample mean for positive/negative stimuli (as determined in Study 1)] or low trial counts (<75% trials) in the unipolar task. Although these quality control thresholds resulted in a relatively high number of exclusions, this is consistent with prior work on online samples (see Limitations section). The remaining 402 participants with acceptable data for both tasks consisted of 206 men, 190 women, 5 non-binary individuals and 1 person who preferred not to answer, aged 19–64 years [mean (SD) = 37.5 years (11.4)]. Participants reported their race as 8 American Indian/Alaskan Native, 98 Asian/Asian American, 106 Black/African American, 1 Native Hawaiian/Pacific Islander, 164 White/European American, and 25 Other/Mixed, and their ethnicity as 108 Hispanic/Latinx and 294 not Hispanic/Latinx.

#### 3.1.2 Stimuli and procedure

As in Study 1, stimuli included emotional facial expressions, scenes, and words, consisting of clearly valenced and ambiguous trials. In Study 2, however, the faces and scenes stimuli were selected from different databases (see Supplemetnary Table S6 for a complete list of stimuli). The faces were drawn from the RADIATE stimulus dataset (Conley et al., 2018), which consisted of models who reported their race/ethnicity as Asian, Black, Hispanic, or White, displaying happy, angry, or surprised expressions. The scenes were drawn from several affective image databases: IAPS (Lang et al., 2008), DIRTI (Haberkamp et al., 2017), GAPED (Dan-Glauser and Scherer, 2011), NAPS (Marchewka et al., 2014), and OASIS (Kurdi et al., 2017), with positive, negative, and ambiguous labels defined by the average participant responses and RTs in a pilot study in our lab. As in Study 1, the words were drawn from Harp et al. (2021) with positive, negative, and ambiguous labels defined by the average participant responses in that study.

A notable difference in the methods for Study 2 was that the order of the task versions (2AFC/unipolar) was counterbalanced, as was the order of positive and negative rating blocks within the unipolar task. This modification was implemented to address the possibility that, in Study 1, participants’ responses in the earlier task could bias their responses to the same stimuli in a subsequent task. Otherwise, the instructions, trial conditions, and response formats were identical to Study 1.

#### 3.1.3 Analyses

Initial analyses were conducted identically to Study 1: Repeated-measures ANOVAs were conducted on average valence bias and unipolar ratings to confirm valence differences between positive, negative, and ambiguous stimuli, and the Spearman’s rank correlation between unipolar positive and negative ratings was examined across participants. Additionally, the minimum value between a participant’s response on the unipolar positive and negative ratings for each stimulus was averaged for each valence category. Next, a linear model predicting 2AFC valence bias was fit with positive and negative unipolar ratings as predictors (preregistered Hypothesis #1); age, sex, race/ethnicity (Asian, Black, Hispanic, and White), and unipolar task order (positive vs. negative ratings first) were included as covariates as in Study 1, as well as task order (2AFC vs. unipolar task first), although these covariates were not specified in the preregistration.

Analyses related to race/ethnicity intergroup differences (preregistered Hypothesis #2) are outside the scope of this replication study, and will be reported separately elsewhere. Participants also completed several self-report questionnaires on mental health and emotion regulation. Surveys included the Beck Depression Inventory (Beck et al., 1961), Emotion Beliefs Questionnaire (Becerra et al., 2020), Emotion Regulation Questionnaire (Gross and John, 2003), Everyday Discrimination Scale (Williams et al., 1997), Need to Belong scale (Leary et al., 2013), Multidimensional Inventory of Black Identity (Sellers et al., 1997), Perceived Stress Scale (Cohen et al., 1983), Range and Differentiation of Emotion Experiences Scale (Kang and Shaver, 2004), State–Trait Anxiety Inventory (Spielberger et al., 1983), and Social Connectedness scale (Lee and Robbins, 1995). Linear models were fit for 2AFC valence bias, unipolar positive ratings, and unipolar negative ratings of ambiguity with the total scores from questionnaires entered as predictors, along with age, sex, and race/ethnicity (preregistered Hypothesis #3). Details on the questionnaires and related results are provided in the Supplementary material.

We report how we determined our sample size, all data exclusions, manipulations, and measures. Data and an analysis script are available on OSF (see text footnote 1). All analyses were conducted and figures created in R Statistical Software, version 4.3.1 (R Core Team, 2023). This study was preregistered on OSF.^3^

### 3.2 Results and discussion

#### 3.2.1 2AFC task

As in Study 1, response categorizations (positive or negative) for each stimulus class (faces, scenes, and words) in the 2AFC task were summarized as the percentage of negative responses (Table 1). As expected, average responses for positive stimuli were lower (more positive) than ambiguous stimuli, which were lower than negative stimuli (*F*(1.85, 929.58) = 7212.16, *p* < 0.001, η = 0.935), demonstrating that the assigned stimulus valence labels were appropriate for the new stimuli in Study 2 as well.

#### 3.2.2 Unipolar task

**Figure 1:**
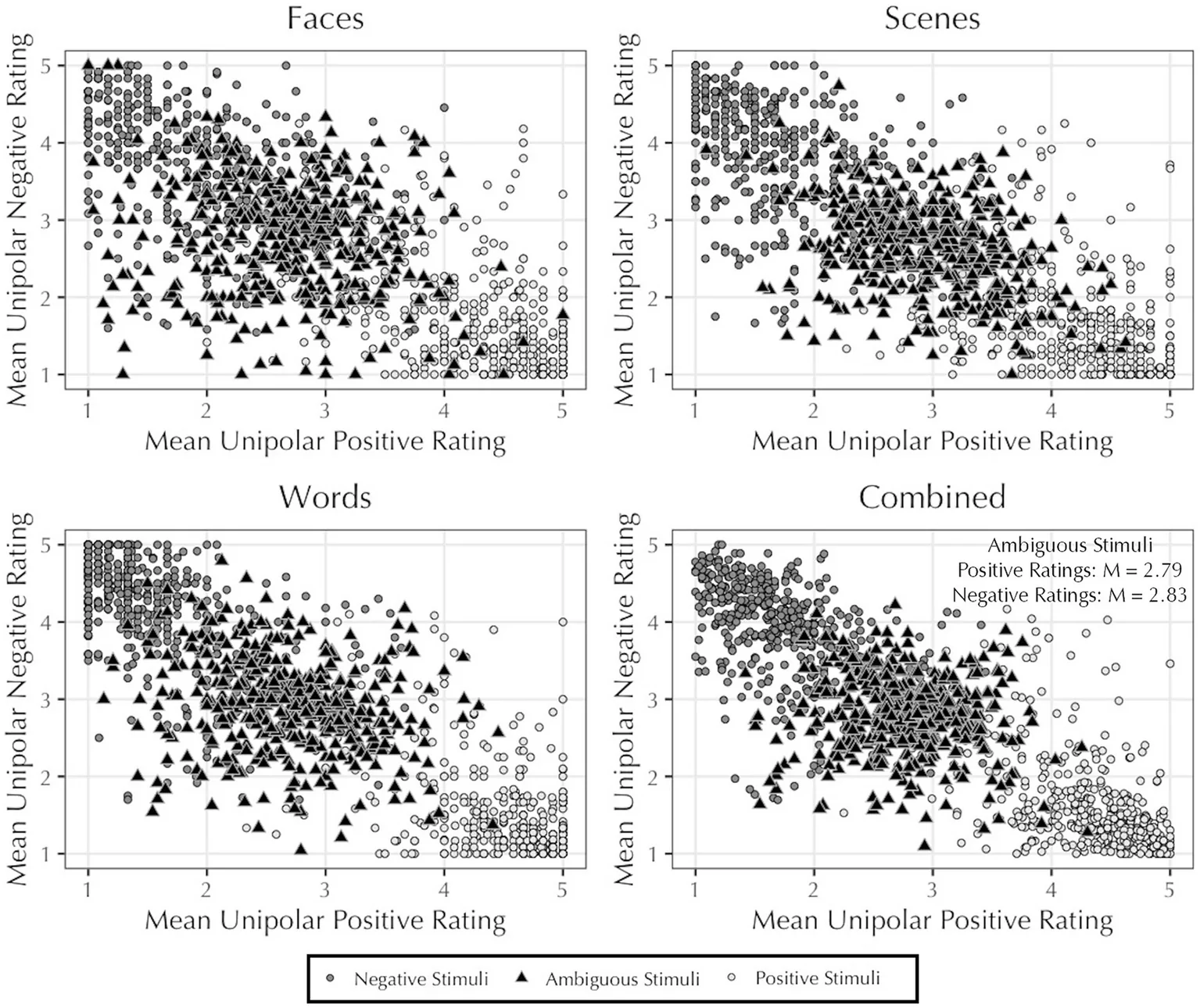
Participants’ mean unipolar positive vs. negative ratings for all stimulus classes separately and combined in Study 2.

Average ratings (possible range 1 to 5) for each stimulus class and valence were calculated for both positive and negative unipolar ratings (Table 1). Overall ratings for the positive and negative scales were calculated as the average of the responses for the three stimulus classes (faces, scenes, and words; Figure 1). As with the 2AFC task, average unipolar positive ratings were higher for positive stimuli than ambiguous stimuli, which were higher than negative stimuli (*F*(1.34, 611.15) = 2929.78, *p* < 0.001, η = 0.866). Conversely, average unipolar negative ratings for positive stimuli were lower than ambiguous stimuli, which were lower than negative stimuli (*F*(1.23, 559.91) = 1478.63, *p* < 0.001, η = 0.765). Participants’ positive and negative unipolar ratings of ambiguous stimuli were negatively correlated across all stimulus classes ( ρ  = − 0.12, 95% CI [−0.22, −0.02], *p* = 0.011) and for each class separately (faces: ρ  = − 0.14, 95% CI [−0.24, −0.04], *p* = 0.003; scenes: ρ  = − 0.31, 95% CI [−0.40, −0.22], *p* < 0.001; words: ρ  = − 0.26, 95% CI [−0.35, −0.16], *p* < 0.001).

The minimum value of the unipolar positive and negative ratings for each stimulus within a participant was averaged for each valence category. For positive stimuli, the average minimum rating was 1.58 (SD = 0.57) and for negative stimuli, the average minimum rating was 1.62 (SD = 0.39). For ambiguous stimuli, the average minimum rating was 1.99 (SD = 0.39), which was significantly higher than either positive (*t*(1203) = 12.79, *p* < 0.001) or negative (*t*(1203) = 11.49, *p* < 0.001) valence stimuli, as in Study 1 (also see Supplementary Figure S3 for a density plot of trial-level responses).

#### 3.2.3 2AFC vs. unipolar responses

**Figure 2:**
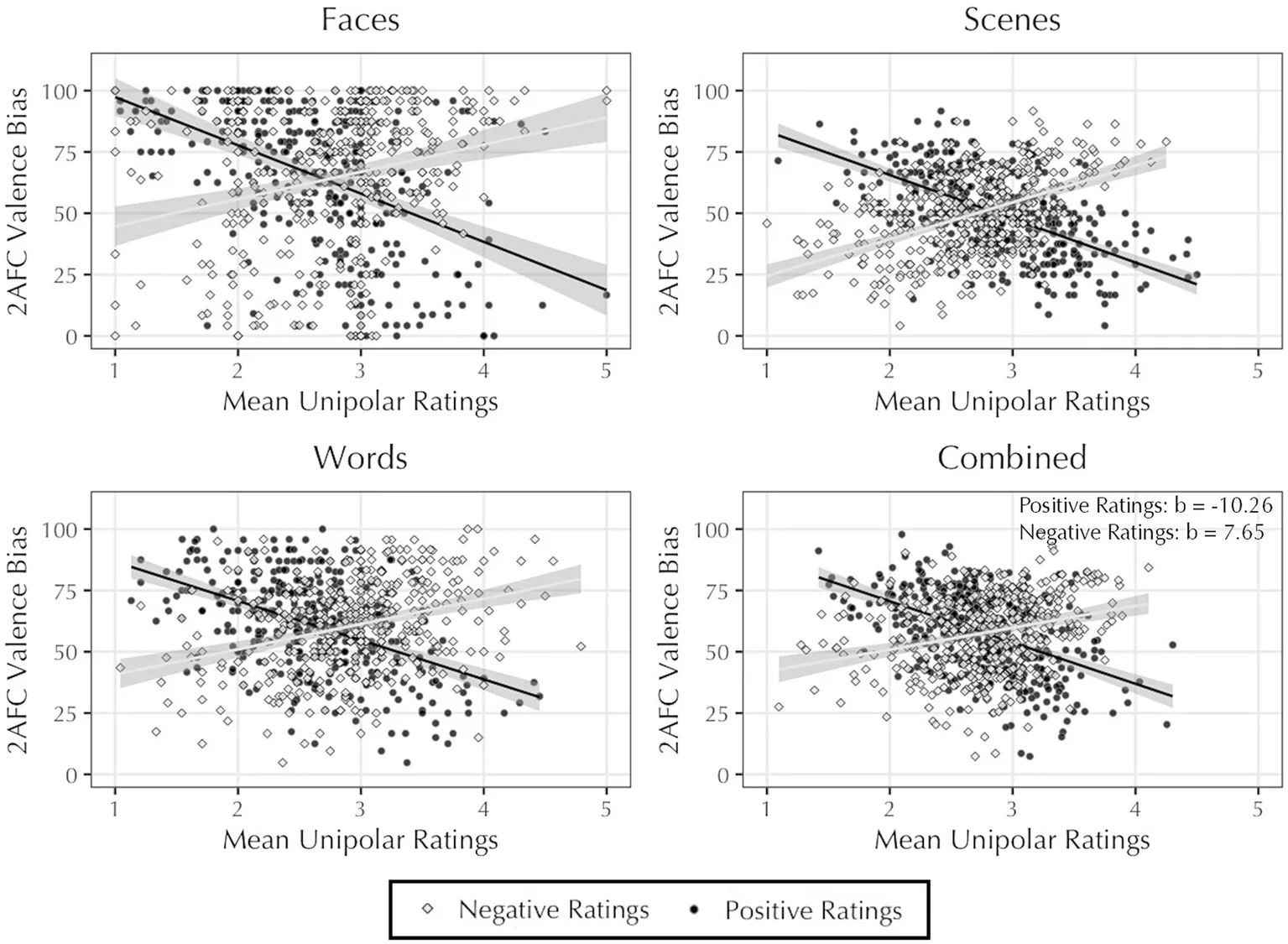
Comparison of 2AFC valence bias (percent negative categorizations) with mean unipolar positive (black circles) and negative (gray diamonds) ratings for each stimulus class individually and combined in Study 2.

To compare responses in the 2AFC and unipolar tasks, a regression model was fit predicting 2AFC valence bias from unipolar positive and negative ratings, using participants’ average ratings across all stimulus classes (i.e., ambiguous faces, scenes, and words). Age, sex, race/ethnicity, task order (2AFC or unipolar task first) and unipolar task order (negative or positive ratings first) were included as covariates. The model was significant overall (*F*(9, 392) = 22.66, *p* < 0.001, *R*^2^ = 0.34) with both unipolar positive and unipolar negative ratings explaining significant variance in 2AFC valence bias (Table 3, Figure 2). Sex, 2AFC/unipolar task order, and positive/negative unipolar task order also reached significance: male participants made more positive 2AFC categorizations than female participants; participants who completed the unipolar task first made more negative 2AFC categorizations, as did participants who completed the negative ratings in the unipolar task before the positive ratings. In addition, a model with only the covariates as predictors was also significant (*F*(7, 394) = 5.12, *p* < 0.001, *R*^2^ = 0.08), such that the change in variance explained by the full model with unipolar ratings was �R ^2^ = 0.26. Models considering each stimulus class separately also showed significant effects of unipolar ratings (see Supplemetnary Tables S10–12).

**Table 3:** Model predicting 2AFC valence bias from unipolar positive and negative ratings in Study 2.

Predictor	*b* (SE)	*t*	*p*	95% CI	*r_a(b.c)_*
Intercept	**82.24 (6.16)**	**13.35**	< 0.001**	**[70.13, 94.35]**	
Unipolar negative	**7.65 (1.38)**	**5.54**	< 0.001**	**[4.93, 10.36]**	**0.229**
Unipolar positive	**−15.51 (1.51)**	**−10.26**	< 0.001**	**[−18.48, −12.54]**	**−0.446**
Age	−0.02 (0.06)	−0.31	0.755	[−0.14, 0.10]	−0.020
Sex (Male)^a^	**−3.02 (1.35)**	**−2.25**	0.025*	**[−5.67, −0.38]**	**−0.087**
Race (Black)^b^	−2.96 (2.00)	−1.48	0.138	[−6.89, 0.96]	−0.064
Race (Hispanic)^b^	−1.66 (1.89)	−0.88	0.379	[−5.37, 2.05]	
Race (White)^b^	−3.76 (1.97)	−1.91	0.057^+^	[−7.64, 0.11]	
2AFC/unipolar task order^c^	**6.27 (1.34)**	**4.68**	< 0.001**	**[3.64, 8.91]**	**0.191**
Unipolar task order^d^	**−3.20 (1.37)**	**−2.34**	0.020*	**[−5.89, −0.51]**	**−0.097**

a Reference = Female.

b Reference = Asian.

c Reference = 2AFC first.

d Reference = negative ratings first.

*r*_a(b.c)_ = semi-partial correlation. Bold font indicates a significant effect: ^+^*p* < 0.10, *p < 0.05, **p < 0.01.

**Figure 3:**
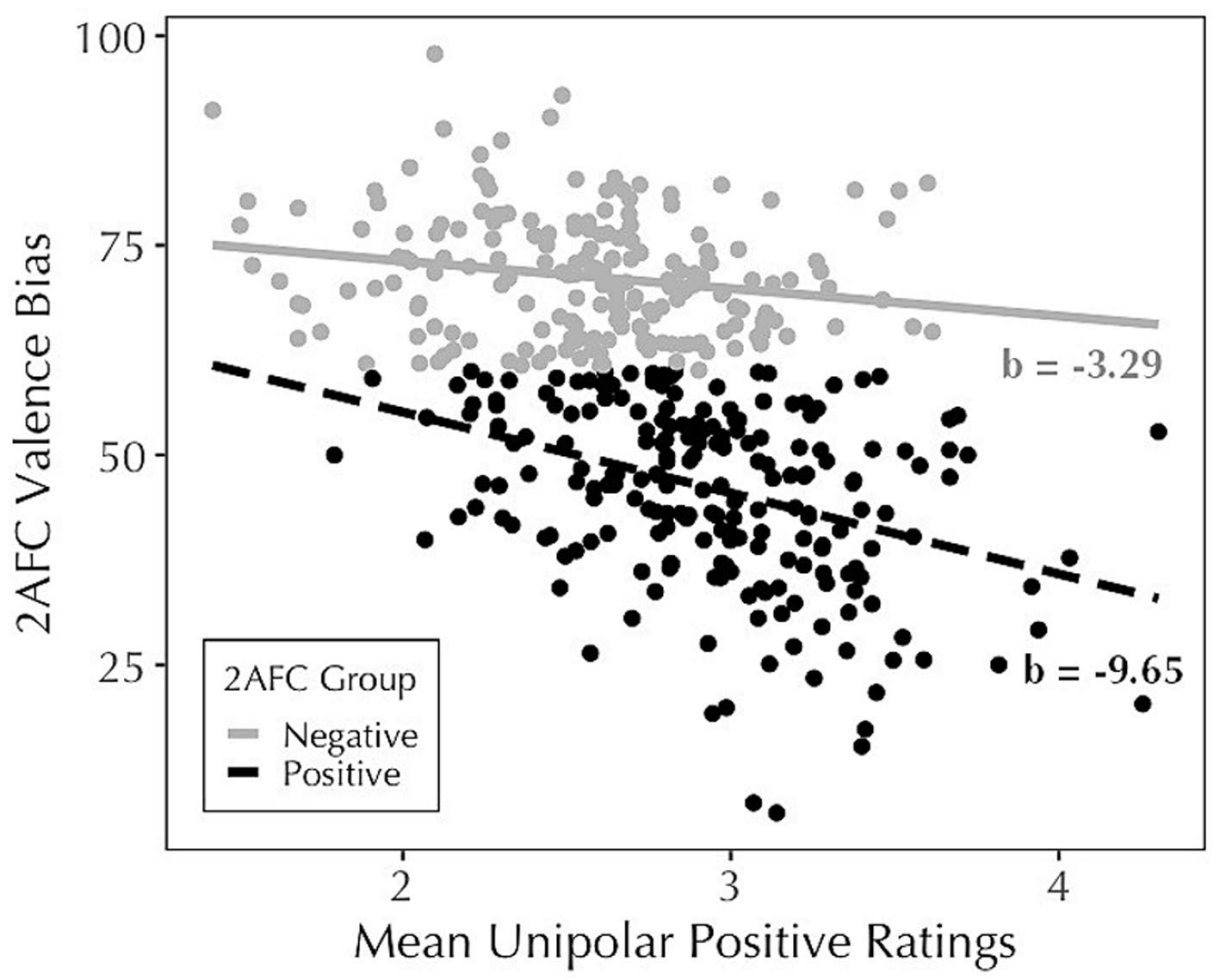
Interaction between 2AFC group (individuals who make mostly negative categorizations vs. mostly positive categorizations) and unipolar positive ratings in predicting valence bias in Study 2. Individuals with a more positive valence bias (*n* = 205) showed a steeper slope between 2AFC and unipolar positive ratings than individuals with a more negative valence bias (*n* = 197).

Given the observed differences in the strength of the effects for unipolar positive and negative ratings on the 2AFC valence bias, a follow-up exploratory model was conducted to further probe the factors that may drive this difference. The model included an additional categorical 2AFC group label [mostly positive judgments (*n* = 205) vs. mostly negative judgments (*n* = 197) defined via median split (59.77%)] as a moderator of unipolar ratings’ effects, as well as RTs for ambiguous trials. The follow-up model was also significant overall (*F*(13, 388) = 76.38, *p* < 0.001, *R*^2^ = 0.72). As above, both unipolar positive and negative ratings showed significant effects, as did 2AFC/unipolar task order and positive/negative unipolar task. Additionally, the effect of ambiguous trial RT and the interaction between 2AFC group and unipolar positive ratings (Figure 3, Supplementary Table S13) were significant. Faster RTs were associated with more negative 2AFC categorizations. For the interaction, a simple slopes analysis indicated that the slope of the effect of unipolar positive ratings on 2AFC categorizations was stronger in the positive 2AFC group (*b* = −9.65, *t* = −6.40, *p* < 0.001) than the negative 2AFC group (*b* = −3.29, *t* = −2.20, *p* = 0.029). The main effects of VB group, age. Sex, race, and the interaction between 2AFC group and unipolar negative ratings were not significant.

#### 3.2.4 Questionnaires

Linear regression models were fit predicting 2AFC valence bias, unipolar positive ratings, or unipolar negative ratings from the self-report questionnaires on mental health and emotion processing. The models for 2AFC valence bias (*F*(15, 444) = 3.56, *p* < 0.001, *R*^2^ = 0.11) and unipolar positive ratings (*F*(16, 399) = 3.89, *p* < 0.001, *R*^2^ = 0.14) were significant overall. Male participants made more positive categorizations than female participants, and Asian participants made more negative categorizations than participants of other racial identities. Greater perceived stress and higher EBQ (belief that emotions are useless/uncontrollable) were associated with more positive categorizations. The unipolar negative ratings model was not significant (*F*(16, 399) = 1.58, *p* = 0.072, *R*^2^ = 0.06). Full details are provided in the Supplementary material.

#### 3.2.5 Summary

The results of Study 2 replicated the findings from Study 1 and demonstrated that these effects extend to new face and scene stimuli with a larger, more diverse participant sample. Across both studies, 2AFC categorizations were related to both positive and negative unipolar ratings, as predicted. These positive and negative unipolar ratings of ambiguous stimuli were moderately negatively correlated, indicating that participants generally treated each scale as the inverse of the other, but not strictly so.

In the unipolar task, participants were willing to separately endorse both positive and negative attributes of the ambiguous stimuli, resulting in average unipolar ratings that were within the middle range of both scales and minimum values that were higher than the values of the clearly valenced stimuli. Ambiguous stimuli may have activated multiple interpretations simultaneously or sequentially, with the valence of the most intense or most prevalent examples driving the final 2AFC response and unipolar ratings. Importantly, the findings from Study 2 showed that these effects were not dependent on the specific stimuli selected for the task or the sample characteristics, but rather reflected a consistent means by which participants approached the type of valence judgments assessed in the present work.

The model comparing unipolar and 2AFC responses indicated that male participants made more positive 2AFC categorizations than females, an effect that has been reported in previous literature on affective biases (Brock et al., 2022; Harp and Neta, 2023; Neta et al., 2019; Norris, 2021). Additional analyses (see Supplementary material) further suggest that these sex differences may arise from lower unipolar negative (but not higher positive) ratings in males, and be present for ambiguous words and scenes, but not faces. Evolutionary or social differences in threat sensitivity may influence how males and females judge emotional stimuli, but additional research is needed to explore the origins of this effect on valence bias.

Finally, we analyzed an exploratory model that examined how the impact of unipolar positive and negative ratings on 2AFC categorizations differs depending upon one’s tendency to be more positive or negative in the 2AFC task. The results from this model indicated that individuals who made more positive 2AFC categorizations showed a stronger effect of unipolar positive ratings on their 2AFC response than individuals who made more negative 2AFC categorizations. The groups did not differ in the effect of unipolar negative ratings. This pattern of results suggests that individuals with a more positive valence bias may be particularly sensitive to possible positive appraisals of ambiguity and preferentially use this information to make their 2AFC decision. This model also yielded a significant RT effect, consistent with prior work (Neta and Tong, 2016; Petro et al., 2021a; Pierce et al., 2023), wherein a more positive 2AFC valence bias was associated with slower responses, potentially reflecting more elaborated processing or reappraisal of the ambiguous stimuli in these individuals (Neta et al., 2021, 2023; Neta and Whalen, 2010).

## 4 General discussion

The two studies presented here demonstrated that valence bias represents individual differences in both perceived positivity and perceived negativity. Our results indicated that both sides of the valence spectrum contributed to the outcome of a seemingly simple categorization choice and that participants recognized the presence of both features in an ambiguous stimulus, rather than relying on only negativity or only positivity. These findings are consistent with prior work demonstrating that individual differences in 2AFC valence bias were associated with variability in both positive and negative daily affect (Puccetti et al., 2023). The standard 2AFC task is therefore a useful measure of variability in appraisals of both positivity and negativity, reflecting judgments of both dimensions of valence in a single measure. In other words, a strong positive valence bias is associated with real positivity in stimulus appraisals, not simply the absence of negativity (and vice versa).

### 4.1 Valence bias arises from both positive and negative appraisals

Prior studies using the 2AFC valence bias task have demonstrated the presence of response competition between the two valence choices using mousetracking data (Brown et al., 2017; Harp et al., 2022; Neta et al., 2021), which can illuminate the decision-making process based on the path of the mouse toward competing responses on the screen. Specifically, when participants made a positive categorization of ambiguity, there was a strong deviation (from a straight path) toward the negative response choice. This was taken as evidence that the initial response instinct is typically negative (Neta and Whalen, 2010; Petro et al., 2018), such that participants began to move the mouse toward this choice on the screen. Subsequently, if the participant engaged cognitive control or reappraisal to overcome this initial negativity (Harp et al., 2024; Neta et al., 2023), then they could shift their motor path to select the positive choice instead. Similarly, other studies have shown that the response conflict elicited by ambiguity slows button-press reaction times regardless of the 2AFC categorization, but especially so for a positive response (Neta and Tong, 2016; Pierce et al., 2023), an effect also observed in the present data.

The current findings supplement prior results by demonstrating that participants typically endorsed both positivity and negativity in their unipolar ratings and that the strength of these ratings drove their 2AFC categorization choice (explaining about 25–50% of the variance in valence bias). Interestingly, across both studies positive unipolar ratings showed larger effect sizes on 2AFC valence bias than did negative unipolar ratings. This might indicate that strong positive interpretations of ambiguity are particularly important for reaching a positive 2AFC decision. This explanation is supported by an exploratory analysis that found individuals with a more positive valence bias exhibited a stronger relationship between unipolar positive ratings and 2AFC responses than individuals with a more negative valence bias; the opposite pattern was not true for unipolar negative ratings. This also aligns with the initial negativity hypothesis of valence bias, which proposes that most young adults initially appraise ambiguous stimuli as negative, as the default interpretation, and that positivity requires additional processing (Neta et al., 2021; Neta and Tong, 2016; Neta and Whalen, 2010). More positive individuals may be uniquely sensitive to positive interpretations of ambiguity and strongly rely on this information, despite also recognizing possible negative interpretations, whereas negative individuals may have limited sensitivity to positive interpretations. Future research could directly assess individual differences in generating valenced appraisals for ambiguous stimuli and explore how these interpretations are integrated during a single trial. Individuals are also slowed by recognition of the conflicting valence of several possible interpretations of ambiguity and those with more similar positive vs. negative unipolar ratings may experience greater conflict.

Some have argued that because surprise does not have a consistent positive or negative interpretation, it might be “valence-free” (Ortony, 2022). Here, we demonstrated that the variability in valence bias is not simply a result of forcing participants to choose between the two alternative valence responses. In the unipolar task, participants could have selected low ratings on both scales for ambiguous stimuli if they were perceived as neutral or representing a state without a specific valence. Instead, emotionally ambiguous stimuli were endorsed as both positive and negative on the unipolar scales, with more positivity than negative stimuli and more negativity than positive stimuli, evidencing their mixed affective value. Furthermore, the average minimum unipolar rating for ambiguous stimuli was greater than for positive or negative stimuli, highlighting that individuals recognized the ambiguity in a single stimulus and that average ambiguity ratings were not driven solely by interindividual differences. The strong relationship between valence bias and unipolar ratings also argues against an interpretation of 2AFC responses as random or guessing, but rather demonstrates that participants are making thoughtful judgments based on the affective content of the stimuli. Thus, the present findings offer clear support for the dual-valence ambiguity of surprised expressions (Neta and Kim, 2023), as well as our selected scenes and words.

It should also be noted that the valence judgments of ambiguity in our tasks utilized different response formats that are known to engage distinct cognitive processes that can influence response characteristics (Hilbert et al., 2016; Rivera-Garrido et al., 2022). This means that participants may have approached the decision differently for the unipolar scales and 2AFC responses—a simple positive/negative categorization can be made quickly and intuitively, whereas a unipolar rating may invoke deeper, more effortful processing to assess the degree of positivity or negativity. In future work, the use of more fine-grained continuous unipolar (i.e., sliding) scales could reveal further distinctions in valence ratings and improve statistical properties of the data, though perhaps also allowing more cognitive influences to mask one’s initial valence bias. The current unipolar task also queried positivity and negativity separately, such that participants may have been encouraged to recognize features of both valence dimensions rather than focusing only on features with the strongest valence to resolve the competition for a binary response. Nonetheless, the significant relationships between the 2AFC and unipolar ratings demonstrate that one’s underlying valence bias systematically influences responses in either format.

### 4.2 Valence bias and well-being

Prior research has demonstrated that 2AFC valence bias is related to emotion regulation (Harp et al., 2024; Neta et al., 2023), emotional nonreactivity (Harp et al., 2022), anxiety (Park et al., 2016), and stress responsivity (Brown et al., 2017; Raio et al., 2021). In the current analyses, we did not find strong evidence of a link between either 2AFC valence bias or unipolar ratings of ambiguity and mental health measures (see Supplementary material). The current findings do, however, help to contextualize previously reported relationships between valence bias and psychological well-being. For instance, a more negative bias has been associated with greater symptoms of depression and negative affect (Neta and Brock, 2021; Petro et al., 2021b), whereas a more positive bias has been associated with greater social connectedness (Neta and Brock, 2021). The present results suggest that this more negative valence bias reflects both an increased tendency toward negativity *and* a decreased tendency toward positivity, situating valence bias at the intersection of positive and negative valence system domains (Morris et al., 2022).

Future longitudinal research could use unipolar ratings to tease apart effects of interventions that shift one’s valence bias. For example, prior work has shown that a mindfulness-based stress reduction (Harp et al., 2022) or cueing of reappraisal (Neta et al., 2023) results in a shift toward more positive/less negative judgments of ambiguity. Future work could determine whether such shifts represent an increase in positive appraisals or a decrease in negative ones, or perhaps both. There may also be other unmeasured variables that relate to unipolar ratings of ambiguously valenced stimuli, such as alexithymia (i.e., difficulty in identifying emotions). Future research could examine additional survey measures and probe how individuals with emotional processing difficulties make valence judgments in different response formats.

### 4.3 Limitations

One limitation of the current work is the presence of significant task order effects – participants were more negative in the 2AFC task when it was completed after the unipolar task. Participants viewed the same stimuli in both tasks in order to compare responses directly while minimizing any cross-task effects of the specific stimuli selected. Given that the average valence bias was more negative than positive, it may be that participants recognized the stimuli or general ambiguity category from the unipolar task and were biased toward making the same valence judgment in the 2AFC task. Nonetheless, task order was counterbalanced across participants and the different response formats required a new type of decision in each task, limiting the likelihood that task order or demand characteristics are driving the relationship between 2AFC and unipolar responses.

Another limitation is the use of online samples, which contributed to a relatively large proportion of participants being excluded from the final analyses. Yet the exclusion rates were similar across the two current studies (Study 1: 39%; Study 2: 35%) and within the expected range (10–50%) based on prior work examining online responses (Barends and de Vries, 2019; Leiner, 2019; Thomas and Clifford, 2017). Our data cleaning procedure (>60% accuracy for clearly valenced stimuli) in the 2AFC task was preregistered and consistent with extensive prior work using this task (Harp et al., 2021; Neta et al., 2013; Neta and Tong, 2016; Petro et al., 2018), ensuring that inattentive or random responses were excluded. The data cleaning procedure for the unipolar task was explored in Study 1, testing different SD thresholds as described in the preregistration. The selected 2 SD threshold resulted in a similar proportion of excluded participants as for the 2AFC task, did not meaningfully change the reported results, and was, therefore, also applied in Study 2.

It is also worth noting that the current tasks assess cognitive judgments and perceptions of ambiguity rather than subjective experiences (Itkes and Kron, 2019). Viewing affective images in a task is likely different from personally experiencing an ambiguous event, during which richer contextual information is typically available. Nonetheless, recognition of others’ emotions and perceptions about ambiguous stimuli are relevant processes for understanding the conceptual structure and experience of ambiguous emotions.

## 5 Conclusion

In conclusion, the present studies provided evidence for the contribution of both positivity and negativity to 2AFC valence judgments of ambiguous stimuli. By combining the relative weights of positive and negative features, participants produced a single valence response that reflected both elements of their appraisal. Despite recognizing the possibility of positive and negative appraisals of ambiguity, individuals with a more negative valence bias may tend to focus on negative features, emphasizing threatening or unpleasant interpretations, while those with a more positive bias focus on more pleasant interpretations, perhaps through an effortful reappraisal process. These tendencies likely persist in everyday experience, with one’s bias toward certain valence appraisals shaping their broader emotion experiences and influencing psychological well-being. Future work should further investigate the dynamics and content of these valenced appraisals of ambiguity, and explore how they are influenced by various real world contexts.

## Data Availability

The datasets presented in this study can be found in online repositories. The names of the repository/repositories and accession number(s) can be found at: https://osf.io/gjv5e.

## Footnotes

1
https://osf.io/gjv5e/

2
www.prolific.com

3
https://osf.io/d2exg/?view_only=1f2b6e4c07274358b75b30f3ef743c34
