# Supplementary material for: Valence bias arises from both positive and negative responses to ambiguous stimuli
Source: Front Psychol. 2026 Aug 26;17:1832043. doi: 10.3389/fpsyg.2026.1832043 (PMC13553364; doi:10.3389/fpsyg.2026.1832043)
Supplement: Supplementary file 1 [file Data_Sheet_1.PDF]

Valence Bias Arises from Both Positive and Negative Responses to Ambiguous Stimuli

Supplemental Material

Jordan E. Pierce, Nicholas R. Harp, James J. Gross, Maital Neta

Prior research has demonstrated that a more negative bias is associated with greater symptoms of depression and anxiety (Neta & Brock, 2021; Park et al., 2016; Petro et al., 2021), more loneliness (Harp & Neta, 2023), increased stress reactivity (Brown et al., 2017; Harp, Freeman, et al., 2022), and lower social connectedness (Neta & Brock, 2021). Conversely, a positive valence bias is associated with greater psychological well-being (Harp, Freeman, et al., 2022). A better understanding of the mechanisms underlying valence bias and how they relate to psychological traits could, therefore, help illuminate how emotion appraisals are generated and how one's typical affective responses contribute to emotional health.

Study 1

In addition to the valence bias tasks, participants completed individual differences measures of psychological well-being and ill-being, emotion regulation tendencies, and beliefs about emotions. We predicted that a negative 2AFC valence bias would be associated with greater psychological ill-being and less frequent cognitive reappraisal, while positive and negative unipolar ratings would uniquely relate to measures of well/ill-being. To anticipate the results, none of these preregistered predicted correlations with psychological traits were found to be significant.

Methods

Stimuli

The stimuli that were presented for each trial in Study 1 are listed in **Table S1** and were identical for the 2AFC and unipolar versions of the task. The face stimuli were selected from the NimStim (Tottenham et al., 2009) and Karolinska Directed Emotional Faces (Lundqvist et al., 1998) databases, the scenes were selected (Neta et al., 2013) from the International Affective Picture System (IAPS; Lang et al., 2008), and the words were taken from a set identified in prior work (Harp et al., 2021).

Table S1. List of the images used in Study 1.

Faces (NimStim; Karolinska)							
**Positive**		**Negative**		**Ambiguous**			
01F_HA_O (N)	36M_HA_O (N)	01F_AN_C (N)	36M_AN_C (N)	01F_SP_O (N)	20M_SP_O (N)	AF01SUS (K)	AM06SUS (K)
03F_HA_O (N)	37M_HA_O (N)	03F_AN_C (N)	37M_AN_C (N)	02F_SP_O (N)	23M_SP_O (N)	AF02SUS (K)	AM12SUS (K)
07F_HA_O (N)	AF06HAS (K)	08F_AN_C (N)	AF07ANS (K)	06F_SP_O (N)	24M_SP_O (N)	AF03SUS (K)	AM13SUS (K)
08F_HA_O (N)	AF08HAS (K)	09F_AN_C (N)	AF14ANS (K)	07F_SP_O (N)	27M_SP_O (N)	AF13SUS (K)	AM18SUS (K)
24M_HA_O (N)	AM01HAS (K)	20M_AN_C (N)	AM10ANS (K)	08F_SP_O (N)	28M_SP_O (N)	AF30SUS (K)	AM34SUS (K)
28M_HA_O (N)	AM14HAS (K)	28M_AN_C (N)	AM28ANS (K)	09F_SP_O (N)	36M_SP_O (N)	AF34SUS (K)	AM35SUS (K)
**Scenes (IAPS)**							
**Positive**		**Negative**		**Ambiguous**			
1440	2550	2717	9421	1030	2688	4598	7590
1441	2340	7380	9424	1303	2704	6410	7620
1460	2347	9050	9432	1560	3211	6837	8010
1710	5825	9185	9561	2339	3310	7430	8060
2057	7502	9220	9830	2480	3360	7460	8466
2091	8380	9295	9910	2485	4233	7570	8501
**Words (Harp et al., 2021)**							
**Positive**		**Negative**		**Ambiguous**			
ACCOMPLISH	HONEY	ILLEGAL	ROTTEN	BATTER	COURT	OPERATION	SHAKE
ATTRACTIVE	LEMONADE	INJURY	SLUM	BEAT	CRUSH	PICK	SNAPPY
CAPABLE	LOVED	KIDNAP	TOXIC	BREAK	DISCIPLINE	RADICAL	SURVIVAL
ENTERTAIN	MOTHER	MAGGOT	UNEASY	CHEAP	FIGHTER	RETREAT	THICK
EXCELLENT	SUNRISE	MORBID	UNSAFE	CHILLING	HAIL	REVOLUTION	TRAFFIC
GIRL	SWEETNESS	PRISON	VILE	CLOSE	HANG	RUSH	TRICK

Questionnaires

Following completion of the 2AFC and unipolar tasks, participants completed several self-report questionnaires on psychological ill-being, well-being, and emotion regulation.

- *Beck Depression Inventory (BDI).* This 20-item scale assesses how the participant has been feeling during the last two weeks (Beck et al., 1961). The question on suicidal thoughts was excluded.
- *Cognitive Flexibility Scale.* This 12-item scale assesses the participant's beliefs about flexibility in their own behavior (Martin & Rubin, 1995).
- *Emotion Beliefs Questionnaire.* This 16-item scale assesses the participant's beliefs about the usefulness and controllability of emotions (Becerra et al., 2020).
- *Emotion Regulation Questionnaire -Frequency (ERQ-FR).* This 10-item scale assesses the participant's *habitual use* of cognitive reappraisal and expressive suppression emotion regulation strategies in daily life (Gross & John, 2003).
- *ERQ-Self-Efficacy (ERQ-SE).* This 10-item scale assesses the participant's beliefs about their *capability* for using cognitive reappraisal and expression suppression strategies in daily life.
- *Eudaimonic Well-being.* This 18-item scale assesses the participant's feelings about themselves and their relationships (Ryff & Keyes, 1995).
- *Peace of Mind.* This 7-item scale assesses how often the participant feels internal peace in their daily life (Y.-C. Lee et al., 2013).
- *Perth Alexithymia Questionnaire.* Although this scale was included in our preregistration as a planned measure, it was not administered to participants.
- *Primals Inventory (PI-18).* This 18-item scale assess the participant's beliefs about the basic character of the world, including being good, safe, enticing, and alive (Clifton & Yaden, 2021).
- *PROMIS Anxiety Short Form 8a.* This 8-item scale assesses how often the participant felt anxious in the past seven days (Pilkonis et al., 2011).

- *Satisfaction with Life Scale.* This 5-item scale assesses the participant's global life satisfaction (Diener et al., 1985).
- *Scale of Positive and Negative Experience (SPANE).* This 12-item scale assesses how often a participant has experienced each feeling during the last four weeks (Diener et al., 2010). Four additional items were added: depressed, anxious, worried, and peaceful.

Analysis

In line with our preregistered analyses, both 2AFC valence bias and unipolar ratings were compared with the self-report measures using Spearman's rank correlations due to a non-normal distribution of valence bias (Shapiro-Wilk test: $W = 0.96$, $p < .05$). We also examined linear models predicting 2AFC valence bias from unipolar ratings for each stimulus class (faces, scenes, words) separately.

Results

Questionnaires

Participants completed survey measures of psychological well-being and ill-being. We predicted that a negative 2AFC valence bias would be associated with greater psychological ill-being and less frequent cognitive reappraisal, while positive and negative unipolar ratings would uniquely relate to measures of well/ill-being. However, we found no significant correlations for any of the preregistered primary or exploratory analyses. These non-significant relationships are shown in **Table S2**.

As with the 2AFC task, we predicted that unipolar task ratings would be related to psychological well-being (positive ratings) and ill-being (negative ratings). Neither positive nor

negative unipolar ratings were related to any of our psychological well-being or ill-being survey measures (**Table S2**).

2AFC vs Unipolar Responses

Regression models were fit predicting 2AFC valence bias from unipolar positive and negative ratings, using participants' average ratings for each stimulus class separately (i.e., faces, scenes, and words), all of which showed similar significant effects as the combined model. The overall models were significant for all three stimulus classes: surprised faces ($F(9, 59) = 5.90$, $p < .001$, $R^2 = .47$), ambiguous scenes ($F(9, 62) = 6.35$, $p < .001$, $R^2 = .48$), and ambiguous words ($F(9, 63) = 7.73$, $p < .001$, $R^2 = .52$). There were significant effects of both unipolar positive and unipolar negative ratings in each model (**Tables S3-5**).

Additionally, regression models were fit predicting 2AFC valence bias from unipolar positive and negative ratings using alternative SD cutoffs for data cleaning. When excluding participants with average ratings for positive and negative stimuli that were more than 1 SD from the group mean, 41 participants remained. The model for this subset of participants was significant overall ($F(9, 31) = 3.36$, $p = .006$, $R^2 = .49$) with a significant effect of unipolar positive ($b = -18.70$, $t = -3.75$, $p < .001$), but not unipolar negative ($b = 8.33$, $t = 1.63$, $p = .114$) ratings. When excluding participants with average ratings for positive and negative stimuli that were more than 3 SD from the group mean, 99 participants remained. The model for this subset of participants was significant overall ($F(9, 89) = 7.59$, $p < .001$, $R^2 = .43$) with significant effects of both unipolar positive ($b = -15.79$, $t = -7.28$, $p < .001$) and unipolar negative ($b = 9.25$, $t = 4.09$, $p < .001$) ratings. While these models demonstrate that the primary results are largely robust to the cutoff value, a 1 SD threshold is overly restrictive and excludes more than half of

the sample and a 3 SD threshold is overly liberal and does not exclude all participants who provided poor ratings for clear valence stimuli (e.g., rated negative scenes as highly positive).

Table S2. Spearman Correlations Between 2AFC Valence Bias, Unipolar Positive/Negative Ratings for Ambiguous Stimuli, and Self-Report Measures

	2AFC Valence Bias (n=100)		Unipolar Positive Ratings (n=75)		Unipolar Negative Ratings (n=75)	
Measure	ρ	*p*-value	ρ	*p*-value	ρ	*p*-value
BDI	-.141	.161	.203	.081^{+}	.052	.657
Cognitive Flexibility	.010	.919	.024	.838	-.134	.252
EBQ General Controllability	.083	.411	-.112	.340	.109	.353
EBQ General Usefulness	.096	.340	-.186	.111	.206	.076^{+}
ERQ FR CR	.010	.918	.040	.734	-.102	.384
ERQ FR ES	-.060	.550	.048	.068^{+}	.060	.607
ERQ SE CR	.027	.790	-.023	.845	-.068	.560
ERQ SE ES	-.038	.706	.047	.691	.058	.622
Eudaimonic Well-being	.052	. 604	-.123	.293	-.129	.269
Peace of Mind	.050	.620	-.064	.585	-.134	.254
PI-18 Good	-.007	.950	.016	.895	-.130	.265
PI-18 Safe	-.007	.953	.003	.981	-.090	.442
PI-18 Enticing	.000	.998	-.074	.526	-.119	.311
PI-18 Alive	.027	.823	.059	.614	-.111	.344
PROMIS Anxiety	-.157	.120	.039	.743	.033	.779
SWLS	.177	. 078^{+}	-.184	.115	.038	.749
SPANE Balance	-.027	.786	-.014	.902	-.048	.685

Note: $^{+}$p<.10. BDI = Beck Depression Inventory; EBQ = Emotion Beliefs Questionnaire; ERQ = Emotion Regulation Questionnaire (FR = Frequency; SE = Self-Efficacy; CR = Cognitive Reappraisal; ES = Expressive Suppression); PI = Primals Inventory; PROMIS = Patient-Reported Outcomes Measurement Information System; SWLS = Satisfaction with Life Scale; SPANE = Scale of Positive and Negative Experience.

Table S3. Model predicting 2AFC judgments of surprised faces from unipolar positive and negative ratings in Study 1.

Predictor	*b* (SE)	*t*	*p*	95% CI	$r_{a(b.c)}$
**Intercept**	**79.58 (20.54)**	**3.87**	**< .001****	**[38.48, 120.69]**	
**Unipolar Negative**	**8.99 (3.95)**	**2.28**	**.026***	**[1.10, 16.89]**	**.218**
**Unipolar Positive**	**-18.41 (3.66)**	**-5.03**	**< .001****	**[-25.73, -11.09]**	**-.490**
Age	0.45 (0.27)	1.68	.099^{+}	[-0.09, 0.98]	.132
Sex[a]	1.19 (5.86)	0.20	.840	[-10.54, 12.92]	-.012
Race (Black)[b]	-4.25 (14.53)	-0.29	.771	[-33.32, 24.82]	
Race (Hispanic)[b]	4.00 (12.63)	0.32	.753	[-21.28, 29.28]	-.123
Race (Mixed/Other)[b]	10.53 (16.51)	0.64	.526	[-22.51, 43.57]	
Race (White)[b]	-8.34 (11.50)	-0.73	.471	[-31.34, 14.66]	
Unipolar Task Order[d]	-3.02 (5.27)	-0.57	.568	[-13.57, 7.52]	-.037

[a]reference = Female; [b]reference = Asian; [c]reference = 2AFC first; [d]reference = negative ratings first; $^{+}p < .10$, * $p < .05$, **$p < .01$

Table S4. Model predicting 2AFC judgments of ambiguous scenes from unipolar positive and negative ratings in Study 1.

Predictor	*b* (SE)	*t*	*p*	95% CI	$r_{a(b.c)}$
**Intercept**	**41.77 (12.24)**	**3.41**	**.001****	**[17.31, 66.23]**	
**Unipolar Negative**	**11.07 (2.48)**	**4.46**	**< .001****	**[6.11, 16.03]**	**.421**
**Unipolar Positive**	**-9.38 (2.58)**	**-3.64**	**< .001****	**[-14.53, -4.23]**	**-.333**
Age	0.005 (0.14)	0.04	.970	[-0.27, 0.28]	.027
Sex[a]	-1.10 (2.72)	-0.41	.686	[-6.53, 4.33]	-.008
Race (Black)[b]	-2.64 (6.60)	-0.40	.691	[-15.83, 10.56]	
Race (Hispanic)[b]	3.84 (6.26)	0.61	.542	[-8.67, 16.36]	-.024
Race (Mixed/Other)[b]	-8.67 (9.23)	-0.94	.351	[-27.11, 9.78]	
Race (White)[b]	-0.20 (5.72)	-0.04	.972	[-11.65, 11.24]	
Unipolar Task Order[d]	0.48 (2.53)	0.19	.849	[-4.58, 5.55]	.032

[a]reference = Female; [b]reference = Asian; [c]reference = 2AFC first; [d]reference = negative ratings first; $^{+}p < .10$, *$p < .05$, **$p < .01$

Table S5. Model predicting 2AFC judgments of ambiguous words from unipolar positive and negative ratings in Study 1.

Predictor	*b* (SE)	*t*	*p*	95% CI	$r_{a(b.c)}$
**Intercept**	**68.62 (16.31)**	**4.21**	**< .001****	**[36.03, 101,22]**	
**Unipolar Negative**	**12.04 (3.32)**	**3.63**	**< .001****	**[5.40, 18.67]**	**.298**
**Unipolar Positive**	**-12.87 (3.06)**	**-4.21**	**< .001****	**[-18.98, -6.76]**	**-.412**
Age	-0.04 (0.17)	-0.22	.825	[-0.38, 0.31]	.002
Sex[a]	-4.20 (3.77)	-1.11	.270	[-11.73, 3.33]	-.092
Race (Black)[b]	-11.14 (9.23)	-1.21	.232	[-29.58, 7.30]	.037
Race (Hispanic)[b]	-13.99 (8.17)	-1.71	$.092^{+}$	[-30.30, 2.33]	
Race (Mixed/Other)[b]	-4.18 (10.89)	-0.38	.702	[-25.94, 17.57]	
Race (White)[b]	-6.63 (7.33)	-0.90	.369	[-21.29, 8.03]	
Unipolar Task Order[d]	-0.93 (3.41)	-0.27	.786	[-7.74, 5.88]	-.034

[a]reference = Female; [b]reference = Asian; [c]reference = 2AFC first; [d]reference = negative ratings first; $^{+}p < .10$, $^{*}p < .05$, $^{**}p < .01$

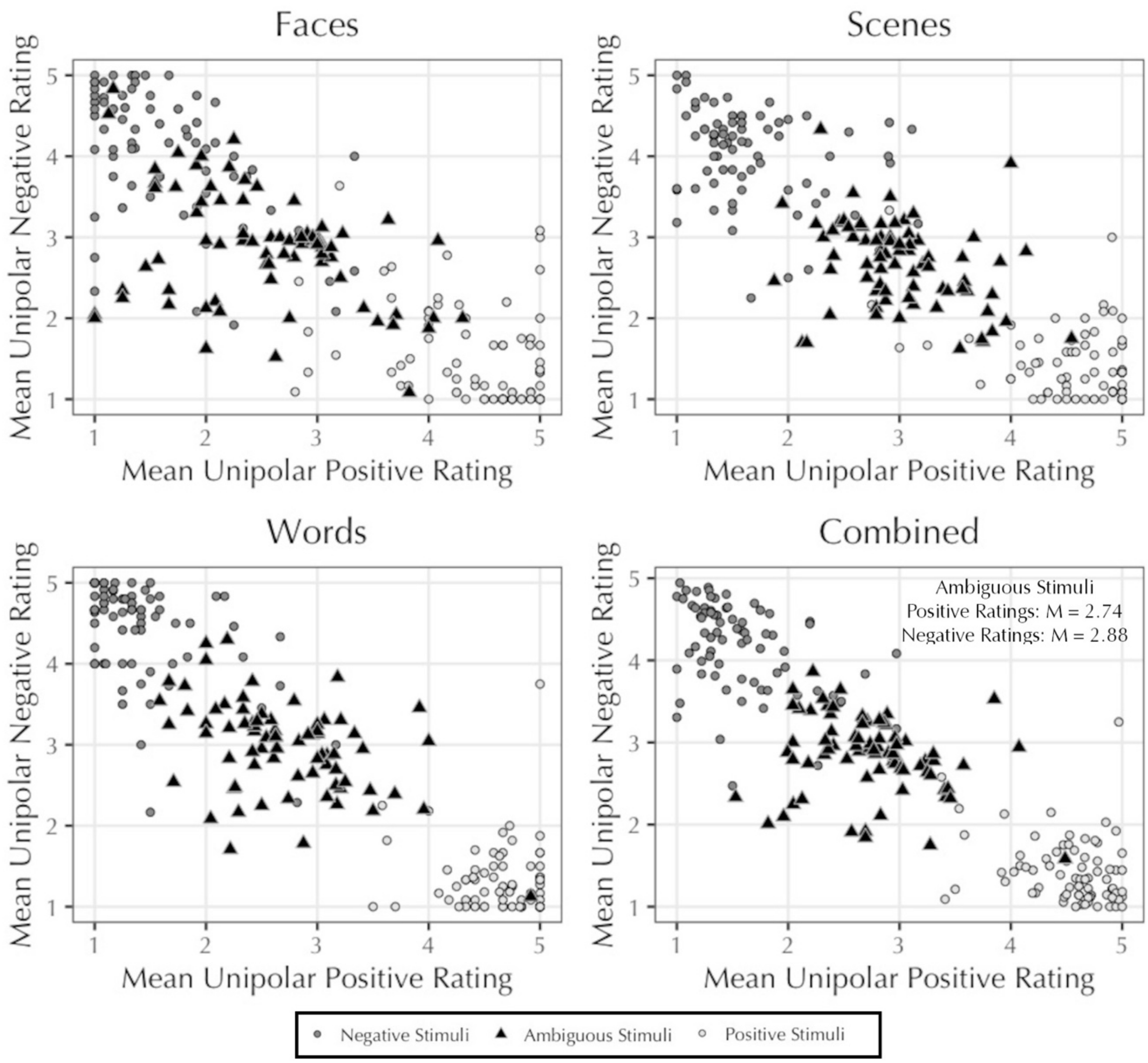

Figure S1 Participants' mean unipolar positive vs negative ratings for all stimulus classes individually and combined in Study 1.

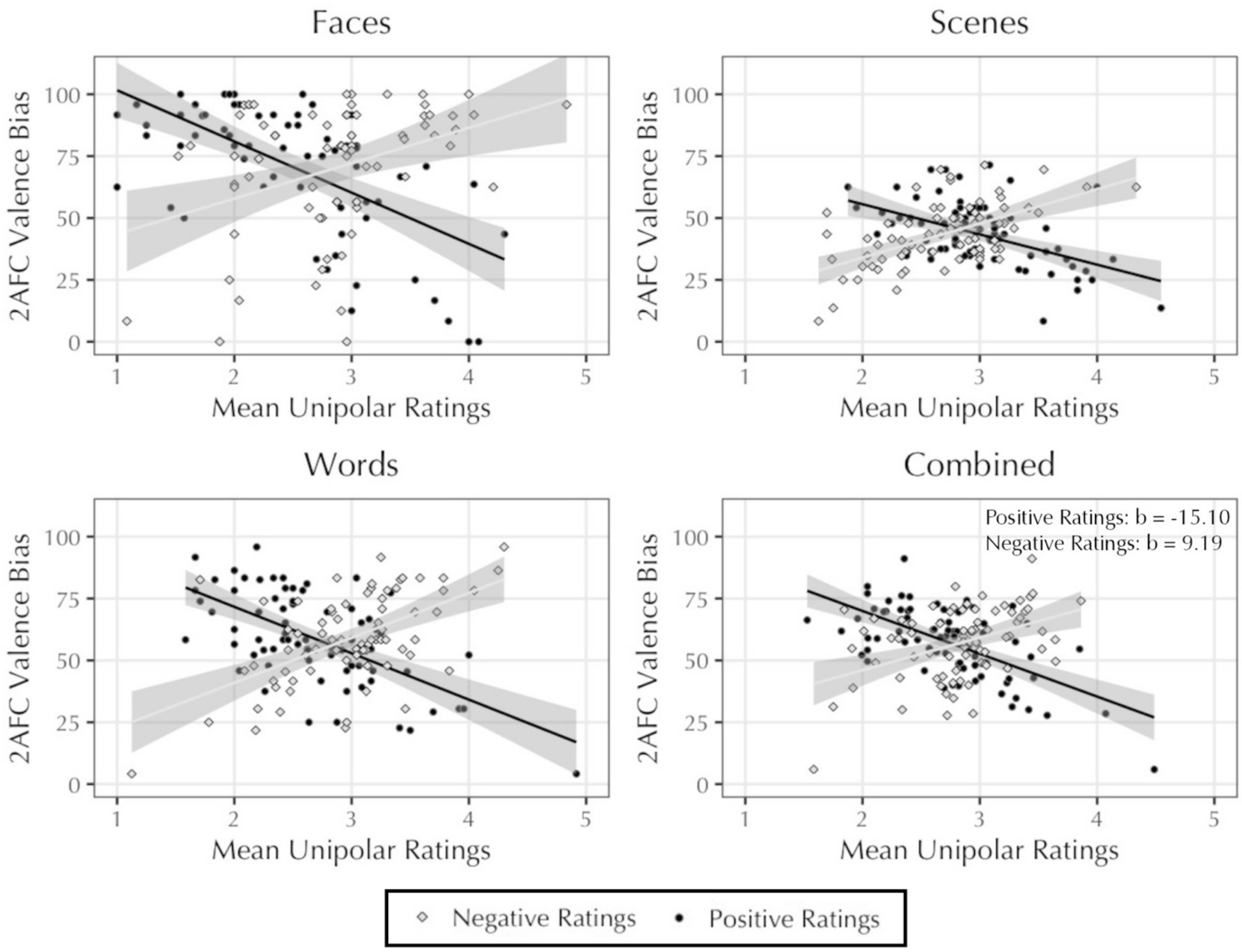

Figure S2 Comparison of 2AFC valence bias (percent negative categorizations) with mean unipolar positive (black circles) and negative (gray diamonds) ratings for each stimulus class individually and combined in Study 1.

Study 2

As in Study 1, following the valence bias tasks participants completed several questionnaires about their mental health, emotion beliefs and regulation tendencies, and experiences of discrimination. We predicted that 2AFC valence bias (i.e., percent negative categorizations of ambiguity) would be positively related to levels of depression (BDI), anxiety (STAI), and stress (PSS) (e.g., more stressful experiences = more negative valence bias). We also predicted that 2AFC valence bias would be negatively related to the tendency to use reappraisal (ERQ-CR), beliefs about the usefulness of emotions (EBQ), emotion differentiation (RDEES), the need to belong, and social connectedness (e.g., more frequent reappraisal = more positive valence bias). Unipolar negative ratings were expected to show effects in the same direction as 2AFC valence bias and unipolar positive ratings were expected to show effects in the opposite direction.

Methods

Stimuli

The stimuli that were presented for each trial in Study 2 are listed in **Table S6** and were identical for the 2AFC and unipolar versions of the task. The face stimuli were selected from the RADIATE (Conley et al., 2018) database, the scenes were selected from several affective image databases: IAPS (Lang et al., 2008), DIRTI (Haberkamp et al., 2017), GAPED (Dan-Glauser & Scherer, 2011), NAPS (Marchewka et al., 2014), and OASIS (Kurdi et al., 2017), and, as in Study 1, the words were taken from a set identified in prior work (Harp et al., 2021).

Table S6. List of the images used in Study 2.

Faces (RADIATE)							
**Positive**		**Negative**		**Ambiguous**			
AF01_HO	HF01_HO	AF02_AO	HF04_AO	AF01_SUR	BF03_SUR	HF01_SUR	WF01_SUR
AF06_HO	HF09_HO	AM03_AO	HM02_AO	AF02_SUR	BF05_SUR	HF04_SUR	WF05_SUR
AM04_HO	HM10_HO	AM07_AO	HM09_AO	AF06_SUR	BF08_SUR	HF09_SUR	WF14_SUR
BF03_HO	WF05_HO	BF05_AO	WF01_AO	AM03_SUR	BM02_SUR	HM02_SUR	WM03_SUR
BM02_HO	WM10_HO	BF08_AO	WF14_AO	AM04_SUR	BM04_SUR	HM09_SUR	WM10_SUR
BM09_HO	WM11_HO	BM04_AO	WM03_AO	AM07_SUR	BM09_SUR	HM10_SUR	WM11_SUR
**Scenes (IAPS, DIRTI, GAPED, NAPS, OASIS)**							
**Positive**		**Negative**		**Ambiguous**			
5725 (I)	P055 (G)	2120 (I)	1078_animals (D)	2092 (I)	H006 (G)	Cemetery_2 (O)	Lion_4 (O)
5829 (I)	Excited_face_5 (O)	2770 (I)	1132_body products (D)	2372 (I)	H013 (G)	Faces_156_h (N)	Objects_037_h (N)
5833 (I)	Nude_couple_7 (O)	9291 (I)	1170_injuries_ infections (D)	9150 (I)	H015 (G)	Faces_260_h (N)	Objects_274_h (N)
5982 (I)	Sunflower_1 (O)	9326 (I)	H007 (G)	9422 (I)	Animals_164_h (N)	Faces_347_h (N)	People_134_h (N)
8467 (I)	Sunset_2 (O)	9471 (I)	Sp089 (G)	A032 (G)	Bee_1 (O)	Landscapes_023_h (N)	Plane_crash_1 (N)
P045 (G)	Yoga_1 (O)	1035_food (D)	Sp137 (G)	A039 (G)	Boxing_1 (O)	Landscapes_045_h (N)	Stingray_1 (O)
**Words (Harp et al., 2021)**							
**Positive**		**Negative**		**Ambiguous**			
ACCOMPLISH	HONEY	ILLEGAL	ROTTEN	BATTER	COURT	OPERATION	SHAKE
ATTRACTIVE	LEMONADE	INJURY	SLUM	BEAT	CRUSH	PICK	SNAPPY
CAPABLE	LOVED	KIDNAP	TOXIC	BREAK	DISCIPLINE	RADICAL	SURVIVAL
ENTERTAIN	MOTHER	MAGGOT	UNEASY	CHEAP	FIGHTER	RETREAT	THICK
EXCELLENT	SUNRISE	MORBID	UNSAFE	CHILLING	HAIL	REVOLUTION	TRAFFIC
GIRL	SWEETNESS	PRISON	VILE	CLOSE	HANG	RUSH	TRICK

Questionnaires

Following completion of the 2AFC and unipolar tasks, participants completed several self-report questionnaires on mental health, emotion regulation, and experiences of discrimination as listed below.

- *Beck Depression Inventory (BDI).* This 20-item scale assesses how the participant has been feeling during the last two weeks (Beck et al., 1961). The question on suicidal thoughts was removed.
- *Demographics.* Participants answered questions about their age, race, ethnicity, gender, and socioeconomic status.
- *Emotion Beliefs Questionnaire (EBQ).* This 16-item scale assesses the participant's beliefs about the usefulness and controllability of emotions (Becerra et al., 2020).
- *Emotion Regulation Questionnaire (ERQ).* This 10-item scale assesses the participant's habitual use of cognitive reappraisal and expressive suppression emotion regulation strategies in daily life (Gross & John, 2003). [ERQ-FR in Study 1.]
- *Everyday Discrimination Scale (EDS)*. This 9-item scale assesses the frequency of the participant's experience with being treated unfairly or disrespectfully in daily life. A follow-up question allows the participant to specify what they believe is the reason for this treatment (e.g., race, religion, disability; Williams et al., 1997).
- *Need to Belong.* This 10-item scale assesses the participant's need for acceptance and belonging in social relationships (Leary et al., 2013).
- *Multidimensional Inventory of Black Identity (MIBI) – Centrality Scale*. This 8-item subscale focuses on the importance of one's racial/ethnic identity to the sense of self

(Sellers et al., 1997). The original wording was modified to “my racial/ethnic group” for use across multiple identities.

- *Perceived Stress Scale (PSS).* This 10-item scale assesses how stressed, upset, or angry the participant has felt about their life in the last month (Cohen et al., 1983).
- *Range and Differentiation of Emotion Experiences Scale (RDEES).* This 14-item scale assesses individual differences in the complexity of emotional experience, with factors focused on the *range* of the participant’s emotionality and their ability to *differentiate* between similar emotions (Kang & Shaver, 2004).
- *State-Trait Anxiety Inventory (STAI).* This 40-item scale assesses how worried or content the participant feels at the current moment and in general (Spielberger et al., 1983).
- *Social Connectedness.* This 8-item scale measures the participant’s sense of belongingness, companionship, and affiliation with others (R. M. Lee & Robbins, 1995).

Analysis

Linear models were fit for 2AFC valence bias, unipolar positive ratings, and unipolar negative ratings of ambiguity with the total scores from each questionnaire entered as predictors, along with age, sex, and race. The EDS and MIBI questionnaires were not included in this analysis and will be considered separately elsewhere. We also examined linear models predicting 2AFC valence bias from unipolar ratings for each stimulus class (faces, scenes, words) separately.

Results

Questionnaires

Linear regression models were fit predicting 2AFC valence bias, unipolar positive ratings, or unipolar negative ratings from the self-report questionnaires on mental health and emotion processing. Predictors included BDI, STAI State/Trait, PSS, ERQ Reappraisal/Suppression, Need to Belong, EBQ, RDEES, and Social Connectedness. Age, sex, and race/ethnicity were also included as covariates. The model for the 2AFC valence bias was significant overall ($F(15, 444) = 3.56$, $p < .001$, $R^2 = .11$). Sex, race, PSS, and EBQ total score were significant predictors. Male participants made more positive categorizations than female participants, and Asian participants made more negative categorizations than participants of other racial identities. Greater perceived stress and higher EBQ (belief that emotions are useless/uncontrollable) were associated with more positive categorizations. Details of the model are provided in **Table S7**.

The model for valence bias in the unipolar positive ratings task was also significant overall ($F(16, 399) = 3.89$, $p < .001$, $R^2 = .14$). Race, PSS, EBQ, and STAI-Trait were significant predictors. Consistent with the 2AFC task, Asian participants made lower positive ratings than participants of other racial identities, and higher PSS and EBQ scores were associated with more positivity. Additionally, higher trait anxiety (STAI-T) was associated with lower positive ratings. Details of the model are provided in **Table S8**.

The model for valence bias in the unipolar negative ratings task was not significant overall ($F(16, 399) = 1.58$, $p = .072$, $R^2 = .06$), and details are provided in **Table S9**.

Table S7. Model predicting 2AFC valence bias from mental health questionnaires.

Predictor	Estimate	S.E.	*t*	*p*
**Intercept**	**74.93**	**10.90**	**6.87**	**< .001****
Age	-0.08	0.07	-1.14	.253
**Sex⁺ (Male)**	**-5.51**	**1.59**	**-3.47**	**< .001****
**Race^ (Black)**	**-8.30**	**2.26**	**-3.68**	**< .001****
**Race^ (Hispanic)**	**-4.70**	**2.22**	**-2.12**	**.035***
**Race^ (White)**	**-6.80**	**2.35**	**-2.93**	**.004****
BDI	-0.02	0.13	-0.17	.869
**PSS**	**-0.42**	**0.18**	**-2.30**	**.022***
ERQ-ES	-0.07	0.66	-0.10	.920
ERQ-CR	-0.44	0.78	-0.57	.572
NtB	0.06	0.11	0.52	.600
**EBQ**	**-0.14**	**0.07**	**-2.10**	**.037***
RDEES	-0.49	1.21	-0.40	.689
SocConn	-0.10	0.12	-0.88	.379
STAI-T	0.19	0.15	1.25	.211
STAI-S	0.11	0.11	1.02	.308

Note: + reference Sex = Female, ^ reference Race = Asian, *$p < .05$ ** $p < .01$. BDI = Beck Depression Inventory, PSS = Perceived Stress Scale, ERQ-ES/CR = Emotion Regulation Questionnaire – Expressive Suppression/Cognitive Reappraisal, NtB = Need to Belong, EBQ = Emotion Beliefs Questionnaire, RDEES = Range and Differentiation of Emotion Experiences Scale, SocConn = Social Connectedness, STAI = State/Trait Anxiety Inventory.

Table S8. Model predicting unipolar positive ratings valence bias from mental health questionnaires.

Predictor	Estimate	S.E.	*t*	*p*
**Intercept**	**2.59**	**0.30**	**8.51**	**< .001****
Age	0.003	0.002	1.59	.112
Sex+ (Male)	0.03	0.05	0.70	.485
Sex+ (NA)	0.28	0.32	0.89	.373
**Race^ (Black)**	**0.33**	**0.06**	**5.05**	**< .001****
**Race^ (Hispanic)**	**0.18**	**0.06**	**2.85**	**.004****
**Race^ (White)**	**0.16**	**0.07**	**2.33**	**.021***
BDI	0.002	0.004	0.64	.521
**PSS**	**0.014**	**0.005**	**2.49**	**.013***
ERQ-ES	-0.002	0.02	-0.10	.923
ERQ-CR	0.02	0.02	0.92	.358
NtB	0.001	0.003	0.35	.726
**EBQ**	**0.004**	**0.002**	**2.09**	**.037***
RDEES	-0.03	0.03	-0.82	.413
SocConn	-0.0003	0.003	-0.10	.922
**STAI-T**	**-0.01**	**0.004**	**-2.34**	**.020***
STAI-S	-0.003	0.003	-0.97	.333

Note: + reference Sex = Female, ^ reference Race = Asian, *$p < .05$ ** $p < .01$.

Table S9. Model predicting unipolar negative ratings valence bias from mental health questionnaires.

Predictor	Estimate	S.E.	*t*	*p*
**Intercept**	**2.33**	**0.34**	**6.79**	**< .001****
**Age**	**0.007**	**0.002**	**2.95**	**.003****
**Sex+ (Male)**	**-0.122**	**0.05**	**-2.37**	**.018***
Sex+ (NA)	0.25	0.36	0.69	.491
Race^ (Black)	-0.10	0.07	-1.36	.174
Race^ (Hispanic)	-0.07	0.07	-0.97	.331
Race^ (White)	-0.06	0.08	-0.83	.409
BDI	-0.0004	0.004	-0.09	.927
PSS	-0.0005	0.006	-0.08	.938
ERQ-ES	0.02	0.02	1.02	.310
ERQ-CR	-0.001	0.02	-0.04	.968
NtB	0.006	0.004	1.47	.141
EBQ	0.003	0.002	1.44	.152
RDEES	0.05	0.04	1.35	.178
SocConn	-0.003	0.004	-0.89	.372
STAI-T	-0.0000	0.005	-0.01	.995
STAI-S	-0.002	0.003	-0.50	.619

Note: + reference Sex = Female, ^ reference Race = Asian, *$p < .05$ ** $p < .01$.

2AFC vs Unipolar Responses

Regression models were fit predicting 2AFC categorizations from unipolar positive and negative ratings, using participants' average ratings for each stimulus class separately (i.e., faces, scenes, and words), all of which showed similar significant effects as the combined model. The overall models were significant for all three stimulus classes: surprised faces ($F(9, 335) = 14.83$, $p < .001$, $R^2 = .28$), ambiguous scenes ($F(9, 363) = 34.19$, $p < .001$, $R^2 = .46$), and ambiguous words ($F(9, 379) = 21.68$, $p < .001$, $R^2 = .34$). There were significant effects of both unipolar positive and unipolar negative ratings in each model (**Tables S10-12**). **Table S13** shows the exploratory model predicting average 2AFC valence bias from unipolar ratings with the additional predictors of ambiguous trial reaction time (RT) and 2AFC group (mostly positive categorizations (<60%) vs. mostly negative categorizations (>=60%)).

Table S10. Model predicting 2AFC judgments of surprised faces from unipolar positive and negative ratings in Study 2.

Predictor	*b* (SE)	*t*	*p*	95% CI	$r_{a(b.c)}$
**Intercept**	**92.38 (9.55)**	**9.67**	**< .001****	**[73.59, 111.17]**	
**Unipolar Negative**	**10.06 (2.00)**	**5.03**	**< .001****	**[6.12, 13.99]**	**.233**
**Unipolar Positive**	**-18.21 (2.16)**	**-8.42**	**< .001****	**[-22.46, -13.96]**	**-.401**
Age	-0.09 (0.12)	-0.76	.448	[-0.34, 0.15]	-.033
Sex[a]	-2.26 (2.76)	-0.82	.412	[-7.68, 3.16]	-.039
Race (Black)[b]	-2.78 (3.96)	-0.70	.483	[-10.57, 5.01]	
Race (Hispanic)[b]	-4.88 (3.84)	-1.27	.204	[-12.44, 2.67]	-.057
Race (White)[b]	-4.34 (3.94)	-1.10	.272	[-12.09, 2.67]	
**2AFC/Unipolar Task Order[c]**	**9.88 (2.74)**	**3.60**	**< .001****	**[4.48, 15.27]**	**.165**
**Unipolar Task Order[d]**	**-8.46 (2.78)**	**-3.04**	**.003****	**[-13.93, -2.98]**	**-.142**

[a]reference = Female; [b]reference = Asian; [c]reference = 2AFC first; [d]reference = negative ratings first; $^+p < .10$, $^*p < .05$, $^{**}p < .01$

Table S11. Model predicting 2AFC judgments of ambiguous scenes from unipolar positive and negative ratings in Study 2.

Predictor	*b* (SE)	*t*	*p*	95% CI	$r_{a(b.c)}$
**Intercept**	**66.41 (6.13)**	**10.83**	**< .001****	**[54.35, 78.48]**	
**Unipolar Negative**	**10.40 (1.30)**	**8.02**	**< .001****	**[7.85, 12.96]**	**.308**
**Unipolar Positive**	**-13.63 (1.33)**	**-10.26**	**< .001****	**[-16.24, -11.02]**	**-.408**
Age	-0.06 (0.06)	-1.01	.313	[-0.18, 0.06]	-.048
**Sex[a]**	**-2.98 (1.33)**	**-2.24**	**.026***	**[-5.60, -0.36]**	**-.083**
Race (Black)[b]	-1.92 (1.97)	-0.98	.330	[-5.79, 1.95]	
Race (Hispanic)[b]	-0.55 (1.86)	-0.29	.769	[-4.21, 3.12]	-.041
Race (White)[b]	-2.77 (1.96)	-1.41	.159	[-6.62, 1.09]	
2AFC/Unipolar Task Order[c]	2.50 (1.32)	1.90	.058^{+}	[-0.08, 5.09]	.073
Unipolar Task Order[d]	-2.41 (1.35)	-1.79	.074^{+}	[-5.06, 0.24]	-.069

[a]reference = Female; [b]reference = Asian; [c]reference = 2AFC first; [d]reference = negative ratings first; $^{+}p < .10$, $^{*}p < .05$, $^{**}p < .01$

Table S12. Model predicting 2AFC judgments of ambiguous words from unipolar positive and negative ratings in Study 2.

Predictor	*b* (SE)	*t*	*p*	95% CI	$r_{a(b.c)}$
**Intercept**	**77.57 (6.83)**	**11.35**	**< .001****	**[64.13, 91.01]**	
**Unipolar Negative**	**6.76 (1.45)**	**4.65**	**< .001****	**[3.90, 9.62]**	**.196**
**Unipolar Positive**	**-13.96 (1.53)**	**-9.12**	**< .001****	**[-16.97, -10.95]**	**-.399**
Age	0.004 (0.07)	0.05	.958	[-0.14, 0.15]	-.014
**Sex[a]**	**-5.12 (1.65)**	**-3.10**	**.002****	**[-8.38, -1.87]**	**-.119**
Race (Black)[b]	-3.74 (2.37)	-1.58	.115	[-8.40, 0.91]	
Race (Hispanic)[b]	0.82 (2.27)	0.36	.717	[-3.64, 5.29]	-.026
Race (White)[b]	-3.20 (2.35)	-1.36	.175	[-7.82, 1.42]	
**2AFC/Unipolar Task Order[c]**	**8.65 (1.63)**	**5.32**	**< .001****	**[5.45, 11.85]**	**.221**
Unipolar Task Order[d]	-0.90 (1.65)	-0.54	.587	[-4.15, 2.35]	-.023

[a]reference = Female; [b]reference = Asian; [c]reference = 2AFC first; [d]reference = negative ratings first; $^{+}p < .10$, $^{*}p < .05$, $^{**}p < .01$

Table S13. Exploratory model predicting average 2AFC valence bias from 2AFC group, unipolar positive and negative ratings in Study 2.

Predictor	*b* (SE)	*t*	*p*	95% CI	$r_{a(b.c)}$
**Intercept**	**76.01 (5.66)**	**13.44**	**< .001****	**[64.89, 87.13]**	
**Unipolar Negative**	**2.90 (1.30)**	**2.24**	**.026***	**[0.35, 5.45]**	**.033**
**Unipolar Positive**	**-3.29 (1.50)**	**-2.20**	**.029***	**[-6.23, -0.35]**	**.026**
Age	-0.003 (0.04)	-0.08	.934	[-0.08, 0.08]	-.002
Sex (Male)[a]	-1.41 (0.89)	-1.58	.114	[-3.16, 0.34]	-.043
Race (Black)[b]	0.54 (1.33)	0.41	.683	[-2.07, 3.16]	
Race (Hispanic)[b]	0.41 (1.26)	0.33	.745	[-2.06, 2.88]	.0006
Race (White) [b]	0.09 (1.31)	0.07	.943	[-2.49, 2.67]	
**2AFC/Unipolar Task Order[c]**	**1.84 (0.91)**	**2.02**	**.044***	**[0.05, 3.62]**	**.055**
**Unipolar Task Order[d]**	**-2.01 (0.90)**	**2.24**	**.026***	**[-3.78, -0.24]**	**-.060**
**Ambiguous RT**	**-0.006 (0.003)**	**2.11**	**.035***	**[-0.01, -0.0004]**	**-.056**
2AFC group[e]	-3.37 (7.93)	-0.43	.671	[-18.96, 12.22]	-.013
2AFC Group[e] * Unipolar Negative	-0.69 (1.81)	-0.38	.703	[-4.24, 2.86]	-.009
**2AFC Group[e] * Unipolar Positive**	**-6.36 (2.09)**	**-3.05**	**.002****	**[-10.46, -2.26]**	**-.082**

[a] reference = Female; [b] reference = Asian; [c]reference = 2AFC first; [d]reference = negative ratings first; [e] reference = negative group; Bold font indicates a significant effect: **p* < .05, ***p* < .01

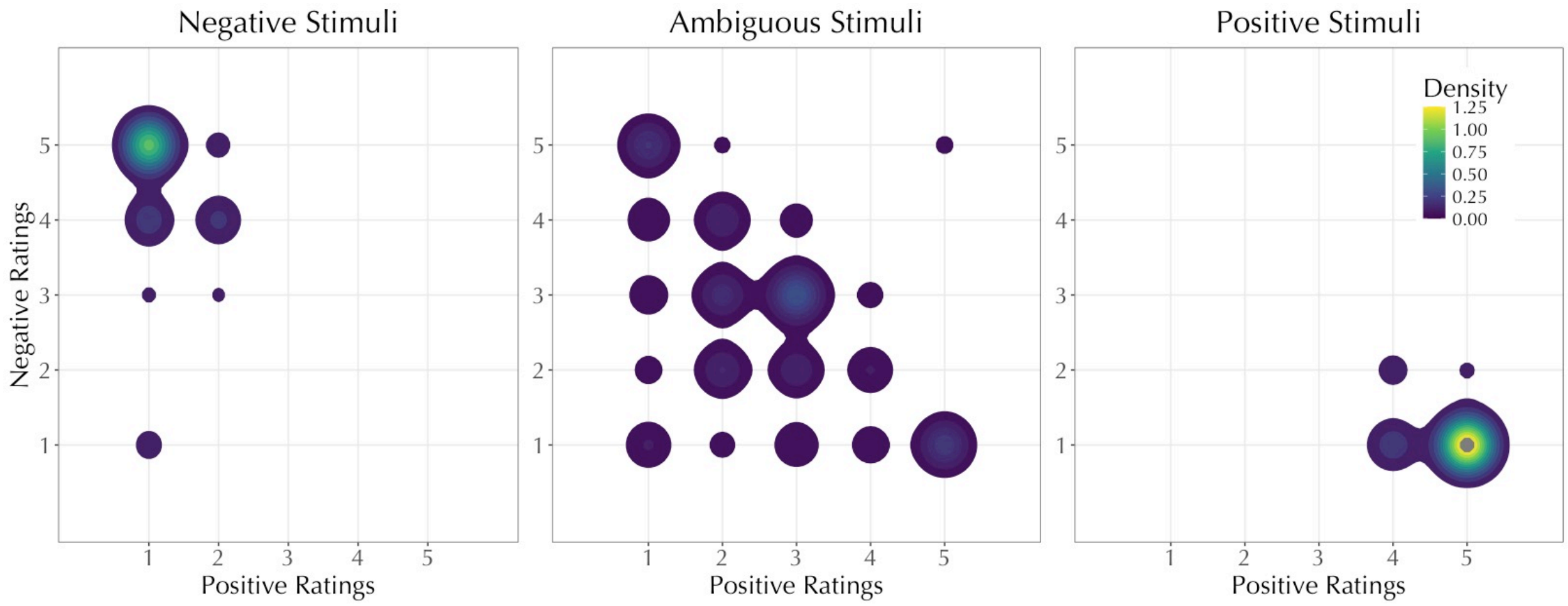

Figure S3. Density plot for individual trial responses in the unipolar task from Study 2. For the clearly valenced conditions, responses were concentrated at high negative/low positive ratings (Negative Stimuli) or high positive/low negative ratings (Positive Stimuli). For the ambiguous conditions, however, responses were more distributed for both unipolar scales with a slight concentration at mid-range positive and negative ratings.

Summary

Overall, the models relating unipolar ratings to 2AFC judgments within each stimulus class showed the same pattern of results as the combined model. More negative 2AFC judgments were associated with higher unipolar negative ratings and lower unipolar positive ratings. Furthermore, there were some significant relationships between 2AFC and unipolar positive ratings with the mental health surveys, although not generally in the expected direction. These models were largely driven by sex and race effects, with smaller effects of stress (PSS) and emotion beliefs (EBQ) in both models. It is unclear why greater perceived stress and a stronger belief that emotions are uncontrollable would be related to greater positivity. Speculatively, a compensatory strategy could be involved in which participants seek to enhance their positive judgments when they feel stressed. On the other hand, for unipolar positive ratings, anxiety (STAI-Trait) did show an effect in the predicted direction: more anxiety was associated with lower positive ratings. In this case, experiencing anxiety symptoms (e.g., feeling worried, insecure) may translate more directly into diminished perceptions of positivity in ambiguous stimuli. Given that these effects were generally weak and inconsistent across studies, further investigation of the relationship between both 2AFC and unipolar ratings of ambiguity and mental health measures is needed.

Discussion

In Study 1, neither 2AFC valence bias nor unipolar ratings were significantly related to any psychological well-being or emotion regulation measures. In Study 2, the models including the psychological health measures significantly predicted 2AFC valence bias and unipolar positive ratings, but not negative unipolar ratings (see supplemental material). Notably, for

unipolar positive ratings, trait anxiety was associated with lower positive ratings of ambiguous stimuli, as expected. This relationship is consistent with prior work (Park et al., 2016), and, again, offers some evidence that positive ratings may make a particularly important contribution to individual differences in affect.

Yet overall, we did not find strong evidence that either 2AFC or unipolar responses to ambiguity were related to psychological traits. There is evidence that the magnitude of the effects of psychosocial measures on valence bias may be small (Harp et al., 2024), such that the current studies may be underpowered to detect these effects. Another possibility is that the implicit bias captured by valence categorizations of ambiguity is poorly reflected by self-report measures. Future work with a larger sample or additional implicit measures could be useful to test these possibilities.

References

Becerra, R., Preece, D. A., & Gross, J. J. (2020). Assessing beliefs about emotions: Development and validation of the Emotion Beliefs Questionnaire. *PLOS ONE*, *15*(4), e0231395. https://doi.org/10.1371/journal.pone.0231395

Beck, A. T., Ward, C. H., Mendelson, M., Mock, J., & Erbaugh, J. (1961). An inventory for measuring depression. *Archives of General Psychiatry*, *4*, 561–571.

Clifton, J. D. W., & Yaden, D. B. (2021). Brief measures of the four highest-order primal world beliefs. *Psychological Assessment*, *33*, 1267–1273. https://doi.org/10.1037/pas0001055

Cohen, S., Kamarck, T., & Mermelstein, R. (1983). A global measure of perceived stress. *Journal of Health and Social Behavior*, 385–396.

Conley, M. I., Dellarco, D. V., Rubien-Thomas, E., Cohen, A. O., Cervera, A., Tottenham, N., & Casey, B. (2018). The racially diverse affective expression (RADIATE) face stimulus set. *Psychiatry Research*, *270*, 1059–1067.

Dan-Glauser, E. S., & Scherer, K. R. (2011). The Geneva affective picture database (GAPED): A new 730-picture database focusing on valence and normative significance. *Behavior Research Methods*, *43*, 468–477.

Diener, E., Emmons, R. A., Larsen, R. J., & Griffin, S. (1985). The Satisfaction With Life Scale. *Journal of Personality Assessment*, *49*(1), 71–75. https://doi.org/10.1207/s15327752jpa4901_13

Diener, E., Wirtz, D., Tov, W., Kim-Prieto, C., Choi, D., Oishi, S., & Biswas-Diener, R. (2010). New Well-being Measures: Short Scales to Assess Flourishing and Positive and Negative Feelings. *Social Indicators Research*, *97*(2), 143–156. https://doi.org/10.1007/s11205-009-9493-y

Gross, J. J., & John, O. P. (2003). Individual differences in two emotion regulation processes: Implications for affect, relationships, and well-being. *Journal of Personality and Social Psychology*, *85*(2), 348.

Haberkamp, A., Glombiewski, J. A., Schmidt, F., & Barke, A. (2017). The DIsgust-RelaTed-Images (DIRTI) database: Validation of a novel standardized set of disgust pictures. *Behaviour Research and Therapy*, *89*, 86–94.

Harp, N. R., Brown, C. C., & Neta, M. (2021). Spring break or heart break? Extending valence bias to emotional words. *Social Psychological and Personality Science*, *12*(7), 1392–1401. https://doi.org/10.1177/1948550620972296

Harp, N. R., Gross, J. J., Uusberg, A., & Neta, M. (2024). The role of trait reappraisal in response to emotional ambiguity: A systematic review and meta-analysis. *Emotion (Washington, D.C.)*, *24*(4), 935–946. https://doi.org/10.1037/emo0001316

Kang, S.-M., & Shaver, P. R. (2004). Individual Differences in Emotional Complexity: Their Psychological Implications. *Journal of Personality*, *72*(4), 687–726. https://doi.org/10.1111/j.0022-3506.2004.00277.x

Kurdi, B., Lozano, S., & Banaji, M. R. (2017). Introducing the Open Affective Standardized Image Set (OASIS). *Behavior Research Methods*, *49*(2), 457–470. https://doi.org/10.3758/s13428-016-0715-3

Lang, P. J., Bradley, M. M., & Cuthbert, B. N. (2008). *International affective picture system (IAPS): Affective ratings of pictures and instruction manual.* Technical Report A-8. University of Florida, Gainesville, FL.

Leary, M. R., Kelly, K. M., Cottrell, C. A., & Schreindorfer, L. S. (2013). Construct validity of the need to belong scale: Mapping the nomological network. *Journal of Personality Assessment*, *95*(6), 610–624. https://doi.org/10.1080/00223891.2013.819511

Lee, R. M., & Robbins, S. B. (1995). Measuring belongingness: The social connectedness and the social assurance scales. *Journal of Counseling Psychology*, *42*(2), 232.

Lee, Y.-C., Lin, Y.-C., Huang, C.-L., & Fredrickson, B. L. (2013). The Construct and Measurement of Peace of Mind. *Journal of Happiness Studies*, *14*(2), 571–590. https://doi.org/10.1007/s10902-012-9343-5

Lundqvist, D., Flykt, A., & Öhman, A. (1998). *The Karolinska Directed Emotional Faces—KDEF (CD ROM).* Stockholm: Karolinska Institute, Department of Clinical Neuroscience, Psychology Section.

Marchewka, A., Żurawski, Ł., Jednoróg, K., & Grabowska, A. (2014). The Nencki Affective Picture System (NAPS): Introduction to a novel, standardized, wide-range, high-quality, realistic picture database. *Behavior Research Methods*, *46*, 596–610.

Martin, M. M., & Rubin, R. B. (1995). A New Measure of Cognitive Flexibility. *Psychological Reports*, *76*(2), 623–626. https://doi.org/10.2466/pr0.1995.76.2.623

Neta, M., Kelley, W. M., & Whalen, P. J. (2013). Neural responses to ambiguity involve domain-general and domain-specific emotion processing systems. *Journal of Cognitive Neuroscience*, *25*(4), 547–557. https://doi.org/10.1162/jocn_a_00363

Park, G., Vasey, M. W., Kim, G., Hu, D. D., & Thayer, J. F. (2016). Trait anxiety is associated with negative interpretations when resolving valence ambiguity of surprised faces. *Frontiers in Psychology*, *7*. https://www.frontiersin.org/articles/10.3389/fpsyg.2016.01164

Pilkonis, P. A., Choi, S. W., Reise, S. P., Stover, A. M., Riley, W. T., & Cella, D. (2011). Item Banks for Measuring Emotional Distress From the Patient-Reported Outcomes Measurement Information System (PROMIS®): Depression, Anxiety, and Anger. *Assessment*, *18*(3), 263–283. https://doi.org/10.1177/1073191111411667

Ryff, C. D., & Keyes, C. L. M. (1995). The structure of psychological well-being revisited. *Journal of Personality and Social Psychology*, *69*, 719–727. https://doi.org/10.1037/0022-3514.69.4.719

Sellers, R. M., Rowley, S. A., Chavous, T. M., Shelton, J. N., & Smith, M. A. (1997). Multidimensional Inventory of Black Identity: A preliminary investigation of reliability and constuct validity. *Journal of Personality and Social Psychology*, *73*(4), 805.

Spielberger, C. D., Gorsuch, R. L., Lushene, R. L., Vagg, P. R., & Jacobs, G. A. (1983). *Manual for the State-Trait Anxiety Inventory (STAI)*. Consulting Psychologists Press.

Tottenham, N., Tanaka, J. W., Leon, A. C., McCarry, T., Nurse, M., Hare, T. A., Marcus, D. J., Westerlund, A., Casey, B., & Nelson, C. (2009). The NimStim set of facial expressions: Judgments from untrained research participants. *Psychiatry Research*, *168*(3), 242–249. https://doi.org/10.1016/j.psychres.2008.05.006

Williams, D. R., Yu, Y., Jackson, J. S., & Anderson, N. B. (1997). Racial differences in physical and mental health: Socio-economic status, stress and discrimination. *Journal of Health Psychology*, *2*(3), 335–351.
